# Multimodal ultrasound-based nomogram for predicting occult cervical lymph node metastasis in cN0 papillary thyroid carcinoma

**DOI:** 10.1186/s12880-026-02648-x

**Published:** 2026-08-10

**Authors:** Yukang Zhang, Yang Guang, Sen Wang, Wen He, Wei Zhang

**Affiliations:** 1 Department of Ultrasound, Beijing Tiantan Hospital, Capital Medical University, Beijing, 100070 China; 2 Department of Ultrasound, Beijing Electric Power Hospital of State Grid Corporation of China, Beijing, 100073 China

**Keywords:** Papillary thyroid carcinoma, Cervical lymph node metastasis, Multimodal ultrasound, Nomogram, Contrast-enhanced ultrasound, Shear wave elastography

## Abstract

**Objective:**

Occult cervical lymph node metastasis (CLNM) remains a clinically relevant issue in clinically node-negative (cN0) papillary thyroid carcinoma (PTC), while its preoperative prediction remains challenging. This study aimed to develop and internally validate a multimodal ultrasound-based nomogram for individualized prediction of occult CLNM in patients with cN0 PTC.

**Methods:**

This retrospective study included 204 patients with pathologically confirmed cN0 PTC who underwent preoperative conventional ultrasonography (US), superb microvascular imaging (SMI), shear wave elastography (SWE), and contrast-enhanced ultrasound (CEUS) between January 2020 and April 2022. Patients were randomly assigned to training (*n* = 143) and validation (*n* = 61) cohorts. Clinical characteristics and multimodal ultrasound features were analyzed. Candidate predictors were screened using univariate logistic regression and least absolute shrinkage and selection operator (LASSO) regression. Conventional ultrasound and multimodal ultrasound nomograms were constructed and evaluated using receiver operating characteristic analysis, calibration curves, decision curve analysis (DCA), interobserver agreement analysis, and bootstrap internal validation.

**Results:**

The multimodal ultrasound nomogram incorporated 12 predictors derived from conventional US, SWE, and CEUS features. The model achieved bootstrap-corrected AUCs of 0.849 (95% CI: 0.776–0.922) and 0.718 (95% CI: 0.559–0.876) in the training and validation cohorts, respectively, outperforming the conventional ultrasound model. DCA demonstrated greater net clinical benefit of the multimodal model across a broader range of threshold probabilities. Interobserver agreement for included imaging features showed good reproducibility.

**Conclusion:**

The multimodal ultrasound nomogram, integrating peritumoral stiffness heterogeneity and CEUS perfusion parameters, showed acceptable performance for predicting occult CLNM in patients with cN0 PTC, providing incremental value compared with the conventional ultrasound model in this single-center cohort. This model may serve as a potential tool for preoperative risk stratification; however, further external validation in larger multicenter studies is required before clinical application.

**Graphical Abstract:**

**Figure:**
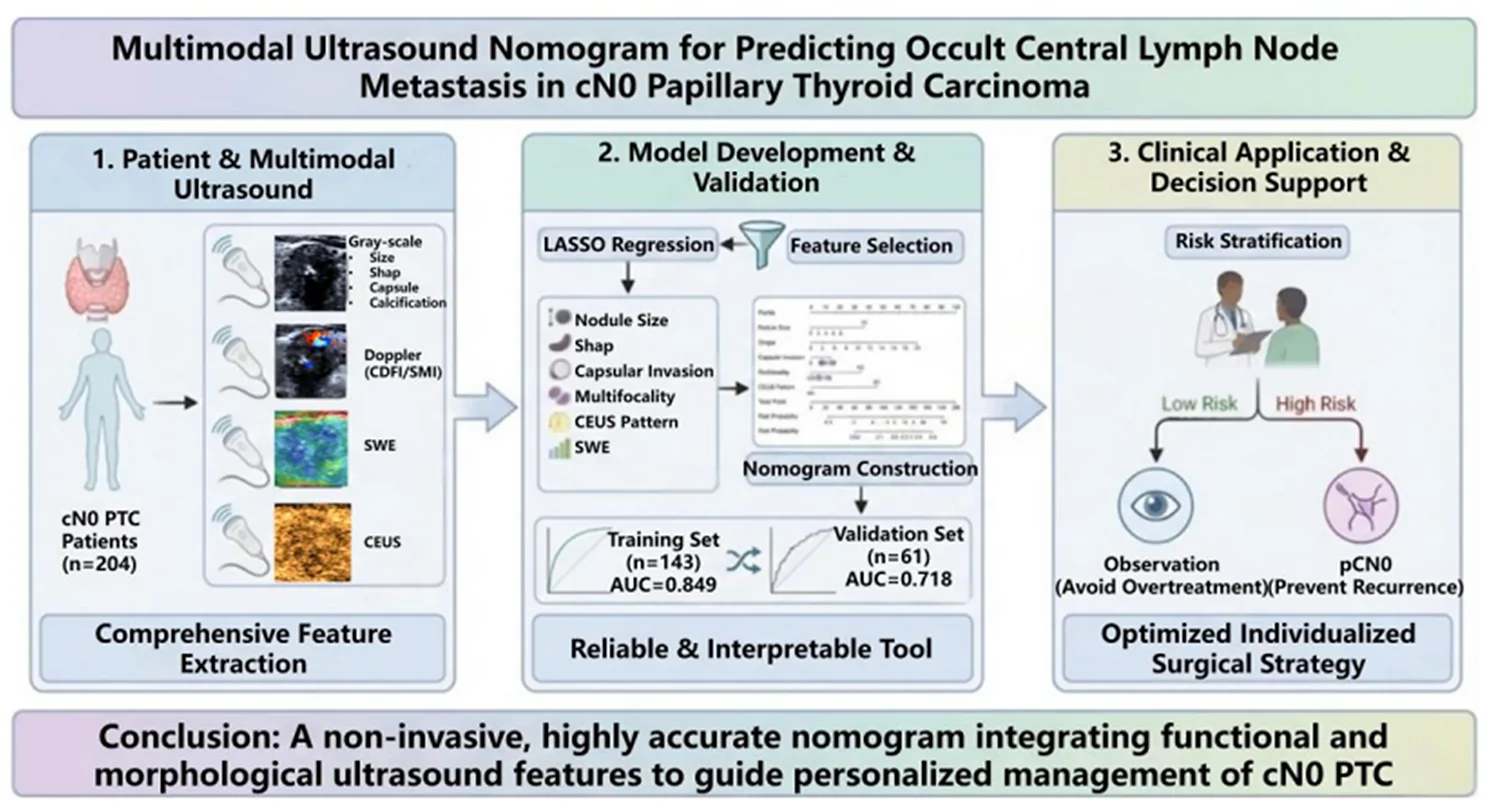

**Supplementary Information:**

The online version contains supplementary material available at https://doi.org/10.1186/s12880-026-02648-x.

## Introduction

Papillary thyroid carcinoma (PTC) is the most common endocrine malignancy. Although PTC generally has an indolent clinical course, cervical lymph node metastasis (CLNM) remains frequent and clinically important [1, 2]. Occult CLNM—defined as pathologically confirmed nodal metastasis despite negative preoperative imaging findings in clinically node-negative (cN0) patients—remains a major challenge for surgical decision-making [3]. Previous studies have reported that the incidence of occult CLNM may exceed 30% even in papillary thyroid microcarcinoma (PTMC), with higher rates noted in larger or high-risk tumors [4]. Therefore, accurate preoperative identification of patients at high risk for occult CLNM is essential to guide individualized management. It may help guide the selective use of prophylactic cervical lymph node dissection, thereby reducing recurrence risk while avoiding unnecessary surgical morbidity in low-risk patients [5].

Conventional ultrasonography (US) remains the primary imaging modality for preoperative risk stratification in PTC. Sonographic features such as microcalcifications and capsular invasion are well-established predictors of CLNM [6]. However, conventional US has limited sensitivity for detecting atypical or deeply located metastatic lymph nodes, while morphological assessment alone may not adequately reflect the biological aggressiveness and heterogeneity of PTC. In recent years, advanced ultrasound techniques, including superb microvascular imaging (SMI), shear wave elastography (SWE), and contrast-enhanced ultrasound (CEUS), have been increasingly applied in clinical practice [7–10]. These modalities provide qualitative and quantitative information on microvascular flow, tissue stiffness, and tumor perfusion. Emerging evidence indicates that these imaging biomarkers are closely associated with tumor aggressiveness and metastatic potential.

Although several studies have investigated multimodal ultrasound-based models for predicting CLNM, further optimization and validation are still needed to establish a comprehensive and clinically applicable model for occult CLNM. Most previous studies have mainly focused on unimodal imaging or qualitative evaluation of thyroid nodules [11–15]. In addition, few studies have systematically assessed the predictive value of peritumoral thyroid gland features, which may indirectly reflect tumor invasiveness and tumor- microenvironment interactions [16–18]. Therefore, developing a practical predictive model that integrates qualitative and quantitative parameters from multimodal ultrasound assessment of both PTC lesions and surrounding thyroid tissue remains clinically significant.

In the present study, we enrolled patients with cN0 PTC and systematically collected preoperative multimodal ultrasound data, including conventional US (grayscale and color Doppler flow imaging [CDFI]), superb microvascular imaging (SMI), shear wave elastography (SWE), and contrast-enhanced ultrasound (CEUS), along with clinical characteristics. Potential predictive factors were screened using univariate logistic regression and least absolute shrinkage and selection operator (LASSO) regression analyses. A nomogram was subsequently constructed and internally validated as a potentially useful tool for individualized preoperative risk assessment in patients with PTC, although further external validation is still required before broader clinical application.

## Patients and methods

### Patients and clinical characteristics

This retrospective cohort study was conducted in accordance with the Declaration of Helsinki and approved by the Institutional Review Board of Beijing Tiantan Hospital, Capital Medical University (Approval No. KY2020-007-02). Clinical records of consecutive patients with cN0 PTC treated between January 2020 and April 2022 were retrospectively reviewed. The requirement for written informed consent was waived due to the retrospective nature of the study and the use of anonymized data.

Patients who underwent preoperative multimodal ultrasound evaluation of thyroid nodules, including grayscale ultrasound, CDFI, SMI, SWE, and CEUS, followed by surgery at Beijing Tiantan Hospital, Capital Medical University between January 2020 and April 2022 were analyzed. Consecutive patients meeting the eligibility criteria were enrolled. The inclusion criteria were as follows: (i) age ≥ 18 years; (ii) for patients with multiple nodules, only the nodule with the highest Thyroid Imaging Reporting and Data System (TI-RADS) category was included; and (iii) histopathologically confirmed PTC after surgery. All patients underwent standardized total thyroidectomy or hemithyroidectomy with at least unilateral prophylactic central neck dissection (pCND) according to institutional protocols. The exclusion criteria were as follows: (i) concurrent head and neck malignancies other than PTC; (ii) previous thyroid surgery; (iii) preoperative ultrasound or computed tomography (CT) findings suggestive of cervical lymph node metastasis (i.e., non-cN0 disease); and (iv) incomplete multimodal ultrasound data.

**Fig. 1:**
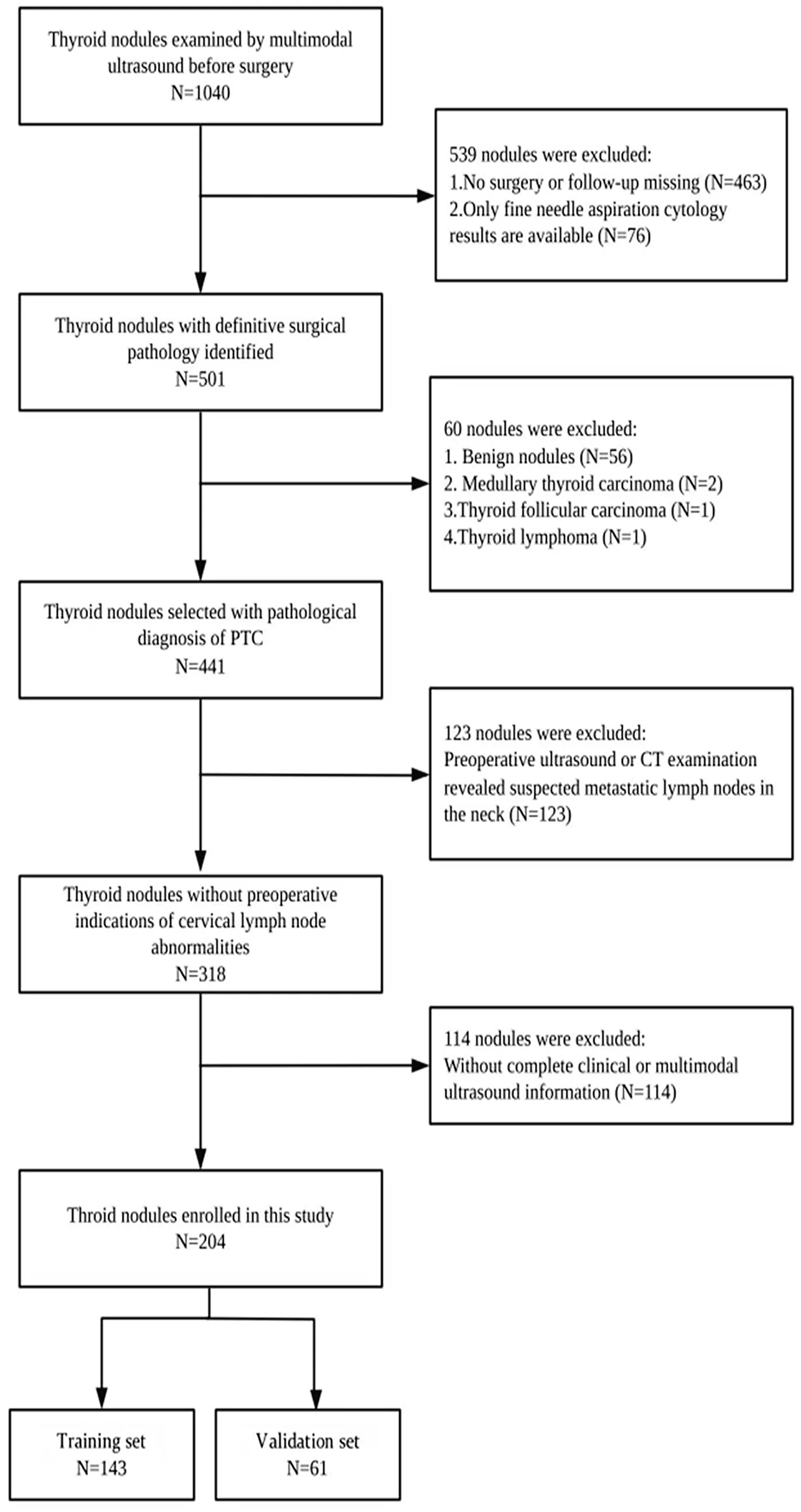
Flowchart of patient enrollment and study design

A total of 1,040 thyroid nodules underwent preoperative multimodal ultrasound evaluation, of which 501 had definitive postoperative histopathological results. After excluding 60 nodules diagnosed as other carcinoma types or benign lesions, 441 nodules were confirmed as PTC. Subsequently, 123 patients with suspected CLNM on preoperative imaging and 114 nodules with incomplete multimodal ultrasound data were excluded. Ultimately, 204 PTC nodules were included and randomly assigned to the training set (*n* = 143) and validation set (*n* = 61) at a 7:3 ratio (Fig. 1).

The following preoperative clinical variables were collected within 1 month before surgery: age, sex, height, weight, and body mass index (BMI).

### Multimodal ultrasound imaging features

All ultrasound assessments were performed using an Aplio i900 ultrasound system (Canon Medical Systems Corporation, Tokyo, Japan) equipped with an i18LX5 linear array probe (frequency range: 5–18 MHz). Patients were positioned supine with the neck slightly extended. Ultrasound examinations were performed by two radiologists with 19 and 11 years of experience in thyroid imaging, respectively. Before the study, both radiologists reviewed and reached consensus on the standardized multimodal ultrasound acquisition and interpretation protocol through a dedicated training process. All examinations followed a predefined standardized multimodal ultrasound protocol described below.

**Fig. 2:**
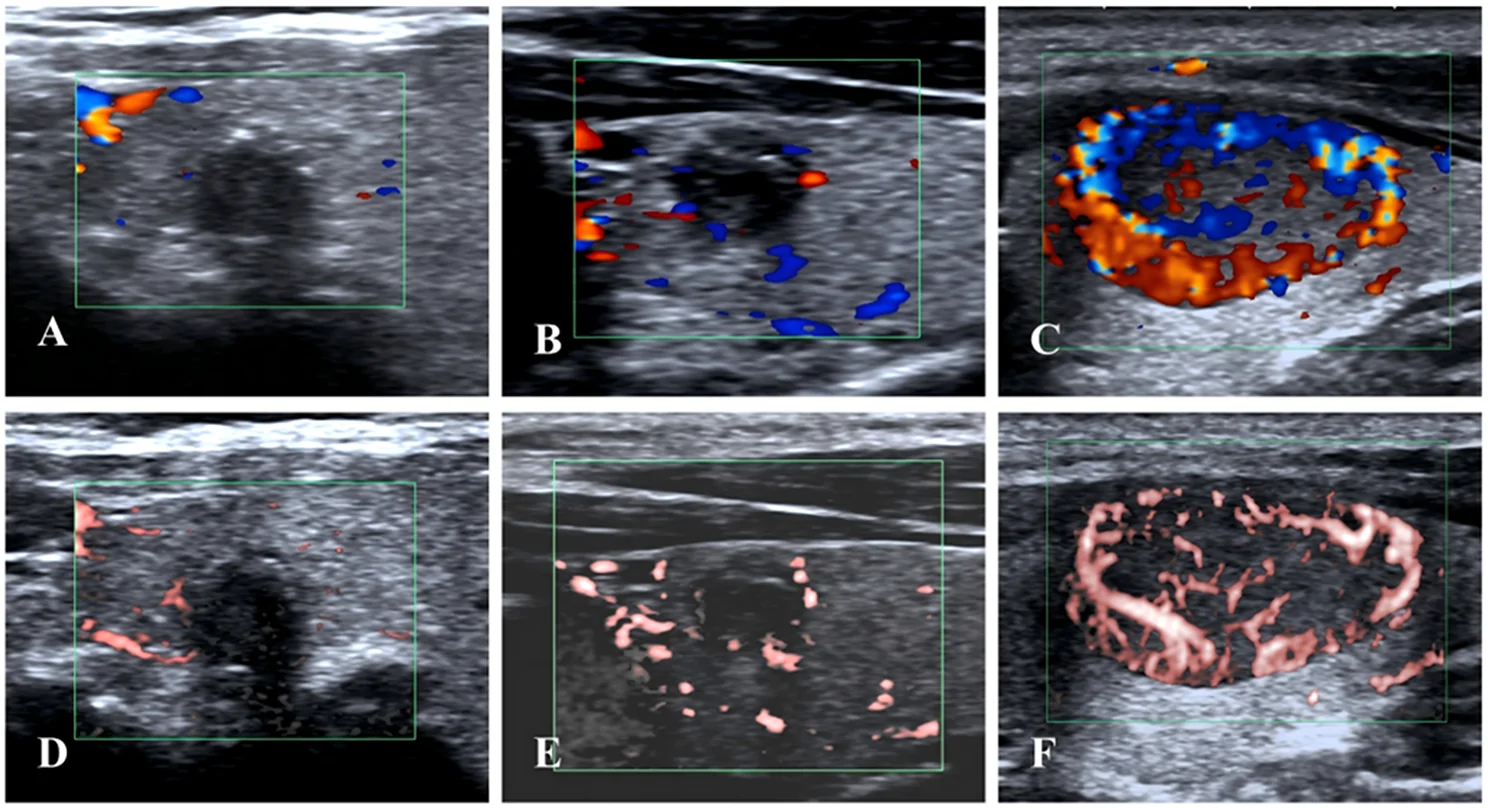
Grading of Color Doppler flow imaging (CDFI) and superb microvascular imaging (SMI) based on the Adler classification. (**A**, **D**) None: no intranodular microvascular flow. (**B**,** E**) Moderate: fewer than 5 punctate or no more than 2 strip-like intranodular blood flow signals. (**C**, **F**) Marked: 5 or more punctate signals or more than 2 major strip-like intranodular blood flow signals

Conventional ultrasound included grayscale ultrasound and CDFI. Nodule multiplicity and location were determined using grayscale ultrasound and postoperative pathology. On grayscale ultrasound, longitudinal, transverse, and anteroposterior diameters were measured. The following features were evaluated: nodule size; margin (well-defined vs. poorly or unable to define); shape (round/oval vs. irregular); echogenicity (hyper/isoechoic vs. hypoechoic); peripheral halo (present or absent); calcification pattern (absent, macrocalcifications, microcalcifications, or mixed-type calcification); microcalcifications quantities (none, < 5, or ≥ 5); taller-than-wide shape (present or absent); relationship between the nodule and thyroid capsule on ultrasound (distant, adjacent/invades the surroundings, or unassessable); capsular contact on ultrasound (< 25%, 25%–50%, or > 50%); and diffuse background parenchymal change (present or absent). Microcalcifications were defined as punctate hyperechoic foci < 1 mm, and mixed-type calcification were defined as the coexistence of macrocalcifications and microcalcifications. CDFI was graded according to the Adler classification: grade I (none), no blood flow signals within or around the nodule; grade II (moderate), fewer than 5 punctate signals or up to 2 linear intranodular flow signals; and grade III (marked), 5 or more punctate signals or more than 2 linear flow signals (Fig. 2A–C) [8]. According to the 2015 American Thyroid Association (ATA) guidelines, conventional ultrasound features suggestive of CLNM included microcalcifications, cystic degeneration, hyperechogenicity, and a round shape [9]. Patients with cervical lymph nodes showing these features were excluded.

SMI was performed in both color and monochrome modes (color SMI, cSMI and Monochrome SMI, mSMI). At the largest cross-sectional plane of the nodule, the lesion contour was manually delineated to define the region of interest (ROI), and the vascular index (VI), defined as the percentage of vascular pixels within the ROI, was automatically calculated. Intranodular vascularity on SMI was graded using the same criteria as CDFI (Fig. 2D–F).

For SWE, circular ROIs (3–5 mm) were placed within the index nodule and adjacent thyroid parenchyma while avoiding calcifications, cystic components, and macroscopic vessels. Three consecutive measurements were obtained to calculate the mean elastic modulus (Eaverage), SD, and the nodule-to-parenchyma elastic modulus ratio.

CEUS was performed using the same ultrasound platform with a mechanical index (MI) of 0.08–0.10. The contrast agent SonoVue (Bracco Imaging, Milan, Italy) was reconstituted with 5 mL sterile saline, and a 1.4–1.6 mL bolus was injected through the antecubital vein, followed by a 5 mL saline flush. Continuous real-time imaging was acquired for 120 s. The following qualitative CEUS features were evaluated: rim definition (well-defined vs. poorly defined); enhancement pattern (centripetal vs. non-centripetal); enhancement intensity (mostly non-enhancement, hypoenhancement, isoenhancement, or hyperenhancement relative to adjacent parenchyma); homogeneity (homogeneous vs. heterogeneous); peripheral enhancing rings (present or absent); relationship with capsule (intact, disrupted, or unassessable); degree of capsular contact (< 25%, 25%–50%, or > 50%); and wash-in/wash-out patterns (earlier than, synchronous with, or later than the surrounding parenchyma). All CEUS clips were digitally stored. Time-intensity curves (TICs) were generated from ROIs placed within the nodule and adjacent thyroid parenchyma. Quantitative perfusion parameters, including arrival time (AT), peak intensity (PI), time to peak (TTP), mean transit time (MTT), area under the TIC (AUTIC), wash-in area (AUWash-in), and wash-out area (AUWash-out), were automatically calculated. Absolute differences between the nodule and adjacent thyroid parenchyma were calculated for each parameter.

After feature extraction, 50 thyroid nodules were randomly selected from the original dataset using a random number table to assess interobserver agreement for the selected ultrasound features. Two radiologists with 10 years of experience in thyroid ultrasound independently re-evaluated these images while blinded to clinical outcomes, pathological results, and each other’s interpretations. The analysis was limited to the ultrasound features ultimately included in the prediction models.

### Statistical Analysis

Missing data were handled using multiple imputation. Kolmogorov–Smirnov test was used for determining whether variables were normally distributed. Normally distributed continuous variables were expressed as mean ± standard deviation and compared using the independent-samples t test, whereas non-normally distributed variables were presented as median (IQR) and compared using the Mann–Whitney U test. Categorical variables were expressed as frequencies and percentages and compared using the chi-square (χ²) or Fisher’s exact test, as appropriate. Variables with *P* < 0.2 in univariate logistic regression were included in a least absolute shrinkage and selection operator (LASSO) logistic regression model for feature selection. The penalty parameter λ was determined by 10-fold cross-validation using the λ.1se criterion. Variables with non-zero coefficients were retained to construct the prediction model and nomogram. To further evaluate the robustness of variables retained by LASSO regression, bootstrap-based stability selection was additionally performed using 1,000 resamples. Variable selection frequency, selection probability, and coefficient sign stability were calculated across bootstrap iterations. Model performance was evaluated in the training and validation sets using the area under the ROC curve (AUC), calibration curves, and decision curve analysis (DCA). In addition, bootstrap internal validation with 500 resamples was performed to further assess model stability and robustness. AUCs were compared using the DeLong test. Interobserver agreement was assessed using Cohen’s Kappa coefficient for dichotomous and nominal variables, weighted Kappa coefficient for ordinal variables, and intraclass correlation coefficient (ICC) for continuous variables. Agreement was classified as excellent (> 0.80), good (0.61–0.80), moderate (0.41–0.60), or poor (≤ 0.40). All statistical analyses were performed using R software (version 4.2.1; R Foundation for Statistical Computing, Vienna, Austria), Free Statistics software (version 1.7.1; Beijing Free Clinical Medical Technology Co., Ltd.), and SPSS software (version 32.0). A two-sided *P* < 0.05 was considered statistically significant. To reduce overfitting, predictor selection was performed using the λ.1se criterion to favor model sparsity and improve generalizability.

## Results

### Comparability of characteristics between CLNM and Non-CLNM groups

Postoperative histopathology confirmed CLNM in 61 patients (29.9%) and no CLNM in 143 patients (70.1%) (Table 1). The mean age was 43.7 ± 11.8 years, and 77.5% were female. No significant intergroup differences were observed in age, height, weight, BMI, or sex (all *P* > 0.05). Multifocality was significantly more prevalent in the CLNM group (50.8%) than in the non-CLNM group (33.6%, *P* = 0.021). Among conventional ultrasound features, the CLNM group showed a larger size (0.9 cm vs. 0.7 cm, *P* < 0.001) and higher rates of irregular shape (95.1% vs. 84.6%, *P* = 0.037), ≥ 5 microcalcifications (49.2% vs. 27.3%, *P* = 0.01), taller-than-wide shape (67.2% vs. 46.2%, *P* = 0.006), adjacency to or invasion of the thyroid capsule (70.5% vs. 47.6%, *P* = 0.003), and > 50% capsular contact (8.2% vs. 3.5%, *P* = 0.009). Diffuse background was more common in the non-CLNM group (47.6% vs. 26.2%, *P* = 0.005). CDFI blood flow grade differed significantly (*P* = 0.014), with a higher proportion of marked flow in the CLNM group (19.7% vs. 7.7%).

**Table 1:** Characteristics between CLNM and Non-CLNM groups / training and validation sets

Variable	Total (*n* = 204)	Non-CLNM and CLNM Groups		*P* value	Training and validation sets		*P* value
		Non-CLNM Group (*n* = 143)	CLNM Group (*n* = 61)		Training set (*n* = 143)	Validation set (*n* = 61)	
**Clinical characteristics**							
Age(years), Mean ± SD	43.7 ± 11.8	44.6 ± 12.1	41.5 ± 10.9	0.083^a^	43.7 ± 11.9	43.7 ± 11.6	0.984^a^
Height(cm), Mean ± SD	163.9 ± 7.9	163.5 ± 8.0	165.0 ± 7.6	0.209^a^	163.9 ± 7.7	164.0 ± 8.3	0.942^a^
Weight(kg), Mean ± SD	67.3 ± 14.2	67.7 ± 14.3	66.4 ± 14.1	0.552^a^	67.8 ± 14.3	66.0 ± 14.0	0.388^a^
BMI, Mean ± SD	24.8 ± 4.3	25.0 ± 4.4	24.2 ± 3.9	0.251^a^	25.0 ± 4.5	24.2 ± 3.6	0.225^a^
Gender, n (%)				0.649^c^			0.411^c^
Female	158 (77.5)	112 (78.3)	46 (75.4)		113 (79)	45 (73.8)	
Male	46 (22.5)	31 (21.7)	15 (24.6)		30 (21)	16 (26.2)	
Location, n (%)				0.103^d^			0.905^d^
Isthmus	9 ( 4.4)	4 (2.8)	5 (8.2)		6 (4.2)	3 (4.9)	
Superior lateral thyroid	40 (19.6)	33 (23.1)	7 (11.5)		27 (18.9)	13 (21.3)	
Middle lateral thyroid	103 (50.5)	71 (49.7)	32 (52.5)		74 (51.7)	29 (47.5)	
Inferior lateral thyroid	52 (25.5)	35 (24.5)	17 (27.9)		36 (25.2)	16 (26.2)	
Multifocality, n (%)				**0.021** ^c^			0.845^c^
Absent	125 (61.3)	95 (66.4)	30 (49.2)		87 (60.8)	38 (62.3)	
Present	79 (38.7)	48 (33.6)	31 (50.8)		56 (39.2)	23 (37.7)	
CLNM, n (%)							0.454^c^
					98 (68.5)	45 (73.8)	
					45 (31.5)	16 (26.2)	
**Conventional ultrasound characteristics**							
Size(cm), Median (IQR)	0.7 (0.6, 1.0)	0.7 (0.6, 0.9)	0.9 (0.7, 1.3)	**< 0.001** ^b^	0.7 (0.6, 1.0)	0.7 (0.6, 1.1)	0.526^b^
Margin, n (%)				0.248^c^			0.825^c^
Well defined	25 (12.3)	20 (14)	5 (8.2)		18 (12.6)	7 (11.5)	
Poorly or unable to define	179 (87.7)	123 (86)	56 (91.8)		125 (87.4)	54 (88.5)	
Shape, n (%)				**0.037** ^c^			0.491^c^
Round or oval	25 (12.3)	22 (15.4)	3 (4.9)		19 (13.3)	6 (9.8)	
Irregular	179 (87.7)	121 (84.6)	58 (95.1)		124 (86.7)	55 (90.2)	
Echogenicity, n (%)				0.727^d^			0.284^d^
Hyperechoic or isoechoic	9 ( 4.4)	7 (4.9)	2 (3.3)		8 (5.6)	1 (1.6)	
Hypoechoic	195 (95.6)	136 (95.1)	59 (96.7)		135 (94.4)	60 (98.4)	
Peripheral halo, n (%)				0.837^c^			0.739^c^
Absent	186 (91.2)	130 (90.9)	56 (91.8)		131 (91.6)	55 (90.2)	
Present	18 ( 8.8)	13 (9.1)	5 (8.2)		12 (8.4)	6 (9.8)	
Calcification, n (%)				0.325^d^			0.305^d^
Absent	79 (38.7)	60 (42)	19 (31.1)		56 (39.2)	23 (37.7)	
Macrocalcifications	3 ( 1.5)	3 (2.1)	0 (0)		1 (0.7)	2 (3.3)	
Microcalcifications	113 (55.4)	74 (51.7)	39 (63.9)		78 (54.5)	35 (57.4)	
mixed-type calcification	9 ( 4.4)	6 (4.2)	3 (4.9)		8 (5.6)	1 (1.6)	
Microcalcifications quantities, n (%)				**0.01** ^c^			0.403^c^
None	82 (40.2)	63 (44.1)	19 (31.1)		57 (39.9)	25 (41)	
< 5	53 (26.0)	41 (28.7)	12 (19.7)		34 (23.8)	19 (31.1)	
≥ 5	69 (33.8)	39 (27.3)	30 (49.2)		52 (36.4)	17 (27.9)	
Taller than wide, n (%)				**0.006** ^c^			0.541^c^
Present	107 (52.5)	66 (46.2)	41 (67.2)		77 (53.8)	30 (49.2)	
Absent	97 (47.5)	77 (53.8)	20 (32.8)		66 (46.2)	31 (50.8)	
US State with capsule, n (%)				**0.003** ^d^			0.258^d^
Distant	87 (42.6)	72 (50.3)	15 (24.6)		62 (43.4)	25 (41)	
Adjacent or invades the surroundings	111 (54.4)	68 (47.6)	43 (70.5)		75 (52.4)	36 (59)	
Unassessable	6 ( 2.9)	3 (2.1)	3 (4.9)		6 (4.2)	0 (0)	
US Contact capsule, n (%)				**0.009** ^d^			0.695^d^
< 25%	152 (74.5)	115 (80.4)	37 (60.7)		104 (72.7)	48 (78.7)	
25% ~ 50%	42 (20.6)	23 (16.1)	19 (31.1)		31 (21.7)	11 (18)	
> 50%	10 ( 4.9)	5 (3.5)	5 (8.2)		8 (5.6)	2 (3.3)	
Diffuse background, n (%)				**0.005** ^c^			0.228^c^
Absent	120 (58.8)	75 (52.4)	45 (73.8)		88 (61.5)	32 (52.5)	
Present	84 (41.2)	68 (47.6)	16 (26.2)		55 (38.5)	29 (47.5)	
CDFI flow grade, n (%)				**0.014** ^c^			0.142^c^
None	82 (40.2)	55 (38.5)	27 (44.3)		63 (44.1)	19 (31.1)	
Moderate	99 (48.5)	77 (53.8)	22 (36.1)		63 (44.1)	36 (59)	
Marked	23 (11.3)	11 (7.7)	12 (19.7)		17 (11.9)	6 (9.8)	
**SMI characteristics**							
SMI flow grade, n (%)				0.717^d^			0.287^d^
None	14 ( 6.9)	9 (6.3)	5 (8.2)		10 (7)	4 (6.6)	
Moderate	124 (60.8)	89 (62.2)	35 (57.4)		82 (57.3)	42 (68.9)	
Marked	66 (32.4)	45 (31.5)	21 (34.4)		51 (35.7)	15 (24.6)	
cSMI- VI (%), Mean ± SD	23.0 ± 14.6	22.5 ± 14.5	24.0 ± 14.9	0.497^a^	23.6 ± 15.8	21.5 ± 11.3	0.341^a^
mSMI- VI (%), Mean ± SD	42.2 ± 21.9	41.9 ± 22.5	43.0 ± 20.7	0.742^a^	41.6 ± 22.7	43.6 ± 20.2	0.571^a^
**SWE characteristics**							
Eaverage- Nodule(m/s), Mean ± SD	3.4 ± 0.9	3.3 ± 0.8	3.5 ± 0.9	0.273^a^	3.4 ± 0.8	3.4 ± 0.9	0.708^a^
Eaverage- Gland(m/s), Mean ± SD	2.4 ± 0.5	2.4 ± 0.5	2.4 ± 0.4	0.496^a^	2.4 ± 0.4	2.4 ± 0.5	0.695^a^
Eaverage-Ratio(m/s), Mean ± SD	1.4 ± 0.4	1.4 ± 0.4	1.5 ± 0.3	0.264^a^	1.4 ± 0.4	1.4 ± 0.4	0.5^a^
SD- Nodule(m/s), Median (IQR)	0.1 (0.1, 0.2)	0.1 (0.1, 0.2)	0.1 (0.1, 0.2)	0.794^b^	0.1 (0.1, 0.2)	0.2 (0.1, 0.2)	0.29^b^
SD- Gland(m/s), Mean ± SD	0.1 ± 0.1	0.1 ± 0.1	0.2 ± 0.1	**< 0.001** ^a^	0.1 ± 0.1	0.1 ± 0.1	0.845^a^
SD- Ratio(m/s), Median (IQR)	1.2 (0.6, 1.9)	1.2 (0.6, 2.0)	1.0 (0.5, 1.7)	0.085^b^	1.1 (0.6, 1.9)	1.4 (0.8, 2.0)	0.127^b^
**CEUS characteristics**							
Rim, n (%)				0.636^d^			**0.029** ^d^
Well defined	5 ( 2.5)	3 (2.1)	2 (3.3)		1 (0.7)	4 (6.6)	
Poorly defined	199 (97.5)	140 (97.9)	59 (96.7)		142 (99.3)	57 (93.4)	
Enhancement pattern, n (%)				**0.002** ^c^			0.955^c^
Centripetal	83 (40.7)	68 (47.6)	15 (24.6)		58 (40.6)	25 (41)	
Non-centripetal	121 (59.3)	75 (52.4)	46 (75.4)		85 (59.4)	36 (59)	
Enhanced intensity, n (%)				0.774^d^			1^d^
Mostly non-enhancement	2 ( 1.0)	1 (0.7)	1 (1.6)		2 (1.4)	0 (0)	
Hypoenhancement	161 (78.9)	113 (79)	48 (78.7)		112 (78.3)	49 (80.3)	
Isoenhancement or hyperenhancement	41 (20.1)	29 (20.3)	12 (19.7)		29 (20.3)	12 (19.7)	
Homogeneity, n (%)				0.774^d^			0.774^d^
Homogeneous	15 ( 7.4)	10 (7)	5 (8.2)		10 (7)	5 (8.2)	
Heterogeneous	189 (92.6)	133 (93)	56 (91.8)		133 (93)	56 (91.8)	
Peripheral enhancement rings, n (%)				0.636^d^			1^d^
Absent	199 (97.5)	140 (97.9)	59 (96.7)		139 (97.2)	60 (98.4)	
Present	5 ( 2.5)	3 (2.1)	2 (3.3)		4 (2.8)	1 (1.6)	
CEUS State with capsule, n (%)				0.688^d^			0.386^d^
Capsule intact	141 (69.1)	100 (69.9)	41 (67.2)		100 (69.9)	41 (67.2)	
Capsule disrupted	59 (28.9)	41 (28.7)	18 (29.5)		39 (27.3)	20 (32.8)	
Unassessable	4 ( 2.0)	2 (1.4)	2 (3.3)		4 (2.8)	0 (0)	
CEUS Contact capsule, n (%)				1^d^			0.143^d^
< 25%	173 (84.8)	121 (84.6)	52 (85.2)		121 (84.6)	52 (85.2)	
25% ~ 50%	24 (11.8)	17 (11.9)	7 (11.5)		15 (10.5)	9 (14.8)	
> 50%	7 ( 3.4)	5 (3.5)	2 (3.3)		7 (4.9)	0 (0)	
Wash in, n (%)				**< 0.001** ^c^			0.943^c^
Earlier	18 ( 8.8)	6 (4.2)	12 (19.7)		13 (9.1)	5 (8.2)	
Synchronous	49 (24.0)	39 (27.3)	10 (16.4)		35 (24.5)	14 (23)	
Later	137 (67.2)	98 (68.5)	39 (63.9)		95 (66.4)	42 (68.9)	
Wash out, n (%)				**0.001** ^d^			0.899^d^
Earlier	135 (66.2)	94 (65.7)	41 (67.2)		93 (65)	42 (68.9)	
Synchronous	53 (26.0)	44 (30.8)	9 (14.8)		38 (26.6)	15 (24.6)	
Later	16 ( 7.8)	5 (3.5)	11 (18)		12 (8.4)	4 (6.6)	
AT- nodule(s), Mean ± SD	11.2 ± 2.5	11.0 ± 2.6	11.4 ± 2.2	0.3^a^	11.1 ± 2.5	11.2 ± 2.5	0.85^a^
AT- gland(s), Mean ± SD	10.3 ± 2.1	10.2 ± 2.1	10.5 ± 2.2	0.385^a^	10.3 ± 2.0	10.3 ± 2.4	0.896^a^
AT- Relative value(s), Median (IQR)	1.0 (0.0, 2.0)	1.0 (0.0, 2.0)	1.0 (0.0, 2.0)	0.743^b^	1.0 (0.0, 2.0)	1.0 (0.0, 2.0)	0.642^b^
PI- Nodule(AU), Median (IQR)	1.4 (0.7, 2.4)	1.4 (0.7, 2.3)	1.4 (0.8, 2.7)	0.542^b^	1.6 (0.8, 2.7)	1.1 (0.6, 2.0)	0.057^b^
PI- Gland(AU), Mean ± SD	3.4 ± 2.3	3.4 ± 2.2	3.5 ± 2.3	0.74^a^	3.5 ± 2.2	3.3 ± 2.3	0.617^a^
PI- Relative value(AU), Median (IQR)	-1.2 (-2.5, -0.4)	-1.2 (-2.5, -0.4)	-1.2 (-2.4, -0.6)	0.8^b^	-1.2 (-2.5, -0.4)	-1.2 (-2.5, -0.6)	0.516^b^
TTP- Nodule(s), Mean ± SD	4.1 ± 1.9	4.1 ± 2.0	4.0 ± 1.5	0.563^a^	4.1 ± 1.9	4.1 ± 1.8	0.933^a^
TTP- Gland(s), Mean ± SD	4.2 ± 1.4	4.2 ± 1.5	4.2 ± 1.2	0.932^a^	4.2 ± 1.6	4.0 ± 1.1	0.286^a^
TTP- Relative value(s), Median (IQR)	-0.2 (-1.0, 0.6)	-0.1 (-1.0, 0.6)	-0.4 (-0.9, 0.5)	0.416^b^	-0.3 (-1.0, 0.5)	0.0 (-0.5, 0.6)	0.126^b^
MTT- Nodule(s), Median (IQR)	7.4 (5.7, 10.6)	7.5 (5.8, 10.1)	7.0 (5.0, 12.3)	0.743^b^	7.2 (5.6, 10.1)	8.0 (6.1, 13.9)	0.065^b^
MTT- Gland(s), Median (IQR)	6.4 (5.3, 8.8)	6.1 (5.1, 8.8)	6.7 (5.7, 8.7)	0.208^b^	6.5 (5.3, 8.6)	6.4 (5.1, 9.7)	0.7^b^
MTT- Relative value(s), Median (IQR)	0.5 (-0.5, 2.6)	0.6 (-0.4, 2.7)	0.5 (-0.8, 2.6)	0.565^b^	0.5 (-0.5, 2.3)	1.0 (-0.4, 2.9)	0.328^b^
AUTIC- Nodule(AU·s), Median (IQR)	0.8 (0.6, 1.5)	0.9 (0.6, 1.6)	0.7 (0.5, 1.4)	0.078^b^	1.0 (0.6, 1.6)	0.8 (0.5, 1.3)	0.076^b^
AUTIC- Gland(AU·s), Median (IQR)	1.9 (1.2, 2.9)	2.0 (1.3, 2.9)	1.6 (1.0, 2.4)	0.112^b^	1.8 (1.2, 2.8)	1.9 (1.0, 3.0)	0.777^b^
AUTIC- Relative value(AU·s), Median (IQR)	-0.6 (-1.6, -0.2)	-0.6 (-1.6, -0.2)	-0.6 (-1.5, -0.3)	0.98^b^	-0.6 (-1.5, -0.2)	-0.7 (-2.0, -0.2)	0.507^b^
AUWash in- Nodule(AU·s), Median (IQR)	0.1 (0.0, 0.1)	0.1 (0.0, 0.1)	0.1 (0.0, 0.1)	0.295^b^	0.1 (0.0, 0.1)	0.0 (0.0, 0.1)	**0.019** ^b^
AUWash in- Gland(AU·s), Median (IQR)	0.2 (0.1, 0.2)	0.2 (0.1, 0.3)	0.1 (0.1, 0.2)	**0.046** ^b^	0.2 (0.1, 0.3)	0.1 (0.1, 0.2)	0.243^b^
AUWash in- Relative value(AU·s), Median (IQR)	-0.1 (-0.2, 0.0)	-0.1 (-0.2, 0.0)	-0.1 (-0.1, 0.0)	0.359^b^	-0.1 (-0.2, 0.0)	-0.1 (-0.2, 0.0)	0.429^b^
AUWash out- Nodule(AU·s), Median (IQR)	0.8 (0.5, 1.4)	0.8 (0.6, 1.4)	0.7 (0.4, 1.3)	0.079^b^	0.8 (0.6, 1.4)	0.8 (0.4, 1.3)	0.116^b^
AUWash out- Gland(AU·s), Median (IQR)	1.7 (1.1, 2.6)	1.8 (1.2, 2.7)	1.5 (0.9, 2.2)	0.084^b^	1.7 (1.2, 2.5)	1.7 (1.0, 2.8)	0.951^b^
AUWash out-Relative value(AU·s), Median (IQR)	-0.6 (-1.4, -0.2)	-0.6 (-1.4, -0.2)	-0.6 (-1.3, -0.2)	0.772^b^	-0.6 (-1.3, -0.2)	-0.7 (-1.9, -0.2)	0.305^b^

Abbreviations: CLNM cervical lymph node metastasis; BMI body mass index; SD standard devistion; IQR interquartile range; US ultrasound; CDFI Color Doppler Flow Imaging; SMI superb microvascular imaging; cSMI-VI color superb microvascular imaging vascular index; mSMI-VI monochrome superb microvascular imaging vascular index; SWE shear wave elastography; CEUS contrast-enhanced ultrasound; AT arrival time; PI peak intensity; AU arbitrary units; TTP time to peak; MTT mean transit time; AUTIC area under the curve; AUWash-in wash-in area; AUWash-out wash-out area

Note: *P*-values with superscript a were calculated by independent-samples t test; *P*-values with superscript b by Mann-Whitney U test; *P*-values with superscript c by Pearson’s Chi-square test; *P*-values with superscript d by Fisher’s exact test

For multimodal ultrasound parameters, no significant differences were observed in SWE-derived nodule stiffness indices between the two groups. However, heterogeneity of adjacent thyroid parenchymal stiffness (SD-Gland) was significantly higher in the CLNM group (*P* < 0.001). On CEUS, the CLNM group more frequently showed a non-centripetal enhancement pattern (75.4% vs. 52.4%, *P* = 0.002), earlier wash-in (19.7% vs. 4.2%, *P* < 0.001), later wash-out (18.0% vs. 3.5%, *P* = 0.001), and lower AUWash-in-gland values (*P* = 0.046). No significant differences were observed in SMI grading or vascular index between groups (all *P* > 0.05).

### Comparability of characteristics between the training and validation sets

The 204 patients were randomly assigned to the training set (*n* = 143) and validation set (*n* = 61) at a 7:3 ratio **(**Table 1**)**. Demographic characteristics, including age, sex, and BMI, were comparable between the two sets (all *P* > 0.05). Most conventional US and multimodal ultrasound features, including shape, echogenicity, calcification pattern, taller-than-wide shape, capsular relationship, and SMI, SWE, and CEUS parameters, were similarly distributed between the two sets. Among qualitative features, only CEUS rim definition differed significantly, with a higher proportion of well-defined rims in the validation set (6.6% vs. 0.7%, *P* = 0.029). No significant differences were observed in SWE or CEUS quantitative parameters except for AUWash-in-Nodule, which was lower in the validation set (*P* = 0.019). The prevalence of CLNM was comparable between the training and validation sets (31.5% vs. 26.2%, *P* = 0.454).

### Univariate analysis of the training set

Univariate logistic regression analysis of clinical and multimodal ultrasound features in the training set (*n* = 143) is summarized in Table 2. Nodule location, multifocality, nodule size, taller-than-wide shape, capsular relationship on ultrasound, degree of capsular contact, diffuse background parenchymal change, SD-Gland, enhancement pattern, wash-in pattern, and wash-out pattern were significantly associated with CLNM. Among clinical and conventional ultrasound features, multifocality (OR = 2.70, 95% CI: 1.31–5.58, *P* = 0.007), nodule size (OR = 1.99, 95% CI: 1.20–3.31, *P* = 0.008), and capsular invasion or adjacency (OR = 3.27, 95% CI: 1.47–7.25, *P* = 0.004) were significant risk factors for CLNM. The risk increased with greater capsular contact (25%–50%: OR = 2.47; >50%: OR = 5.00). Taller-than-wide shape and a non-centripetal enhancement pattern (OR = 2.85, 95% CI: 1.30–6.26, *P* = 0.009) were also associated with higher risk. Regarding CEUS dynamics, synchronous or delayed wash-in (OR = 0.09 and 0.21, respectively) and synchronous wash-out (OR = 0.37) were associated with reduced CLNM risk, whereas delayed wash-out increased risk (OR = 4.00). Tumors located in the superior lateral thyroid (OR = 0.11, 95% CI: 0.02–0.80, *P* = 0.029) and diffuse background parenchymal change (OR = 0.40, 95% CI: 0.18–0.87, *P* = 0.022) showed protective effects. Although SD-Gland was retained in multivariable feature selection, its wide confidence interval in univariate analysis suggested instability, likely due to limited sample size. Other variables, including demographic factors, several conventional ultrasound features, SMI parameters, and most quantitative SWE and CEUS indices, were not significantly associated with CLNM (all *P* > 0.05).

**Table 2:** Univariate logistic analysis of risk fators for CLNM in training group of cN0 PTC patients

Variable	OR(95%CI)	*P* value	Variable	OR(95%CI)	*P* value
**Clinical characteristics**			mSMI- VI (%)	1 (0.99 ~ 1.02)	0.86
Age (years)	0.98 (0.95 ~ 1.01)	0.25	**SWE characteristics**		
Height (cm)	1.02 (0.97 ~ 1.07)	0.43	Eaverage- Nodule(m/s)	1.14 (0.75 ~ 1.74)	0.541
Weight (kg)	0.99 (0.97 ~ 1.02)	0.487	Eaverage- Gland(m/s)	0.57 (0.24 ~ 1.35)	0.202
BMI	0.96 (0.88 ~ 1.04)	0.315	Eaverage-Ratio(m/s)	1.73 (0.68 ~ 4.38)	0.249
Gender			SD- Nodule(m/s)	2.29 (0.19 ~ 26.94)	0.511
Female	1		SD- Gland(m/s)	377.08 (3.82 ~ 37234.52)	**0.011**
Male	0.92 (0.38–2.2)	0.846	SD- Ratio(m/s)	0.82 (0.61 ~ 1.12)	0.219
Location			**CEUS characteristics**		
Isthmus	1		Rim		
Superior lateral thyroid	0.11 (0.02–0.8)	**0.029**	Well defined	1	
Middle lateral thyroid	0.23 (0.04–1.32)	**0.099**	Poorly defined	982660.83 (0 ~ Inf)	0.988
Inferior lateral thyroid	0.28 (0.05–1.76)	**0.176**	Enhancement pattern		
Multifocality			Centripetal	1	
Absent	1		Non-centripetal	2.85 (1.3 ~ 6.26)	**0.009**
Present	2.7 (1.31 ~ 5.58)	**0.007**	Enhanced intensity		
**Conventional ultrasound characteristics**			Mostly non-enhancement	1	
Size(cm)	1.99 (1.2 ~ 3.31)	**0.008**	Hypoenhancement	0.49 (0.03 ~ 8.11)	0.621
Margin			Isoenhancement or hyperenhancement	0.32 (0.02 ~ 5.78)	0.439
Well defined	1		Homogeneity		
Poorly or unable to define	2.53 (0.69–9.23)	**0.16**	Homogeneous	1	
Shape			Heterogeneous	1.08 (0.27 ~ 4.37)	0.917
Round or oval	1		Peripheral enhancement rings		
Irregular	4.51 (1- 20.45)	**0.051**	Absent	1	
Echogenicity			Present	0.72 (0.07 ~ 7.12)	0.778
Hyperechoic or isoechoic	1		CEUS State with capsule		
Hypoechoic	3.38 (0.4 ~ 28.37)	0.261	Capsule intact	1	
Peripheral halo			Capsule disrupted	0.99 (0.44 ~ 2.2)	0.979
Absent			Unassessable	2.23 (0.3 ~ 16.53)	0.434
Present	1.1 (0.31 ~ 3.85)	0.884	CEUS Contact capsule		
Calcification			< 25%	1	
Absent	1		25% ~ 50%	1.09 (0.35 ~ 3.42)	0.88
Macrocalcification	0 (0 ~ Inf)	0.988	> 50%	0.87 (0.16 ~ 4.71)	0.875
Microcalcification	1.25 (0.59 ~ 2.64)	0.558	Wash in		
mixed-type calcification	1.5 (0.32 ~ 7.03)	0.607	Earlier	1	
Microcalcification quantities			Synchronous	0.09 (0.02 ~ 0.4)	**0.001**
None	1		Later	0.21 (0.06 ~ 0.72)	**0.013**
< 5	0.66 (0.24 ~ 1.83)	0.429	Wash out		
≥ 5	1.88 (0.85 ~ 4.17)	**0.121**	Earlier	1	
Taller than wide			Synchronous	0.37 (0.14 ~ 0.99)	**0.048**
Present	1		Later	4 (1.12 ~ 14.32)	**0.033**
Absent	0.46 (0.22 ~ 0.96)	**0.039**	AT- nodule (s)	1.12 (0.97 ~ 1.3)	**0.128**
US State with capsule			AT- gland (s)	1.11 (0.93 ~ 1.32)	0.236
Distant	1		AT- Relative value (s)	1.1 (0.87 ~ 1.39)	0.435
Adjacent or invades the surroundings	3.27 (1.47 ~ 7.25)	**0.004**	PI- Nodule (AU)	0.96 (0.76 ~ 1.22)	0.736
Unassessable	4.64 (0.82 ~ 26.1)	**0.082**	PI- Gland (AU)	0.96 (0.82 ~ 1.13)	0.621
US Contact capsule			PI- Relative value (AU)	1.03 (0.86 ~ 1.23)	0.765
< 25%	1		TTP- Nodule (s)	0.96 (0.79 ~ 1.16)	0.664
25% ~ 50%	2.47 (1.07 ~ 5.69)	**0.034**	TTP- Gland (s)	0.95 (0.75 ~ 1.2)	0.666
> 50%	5 (1.12 ~ 22.38)	**0.035**	TTP- Relative value (s)	0.99 (0.79 ~ 1.23)	0.926
diffuse background			MTT- Nodule (s)	1 (0.93 ~ 1.07)	0.95
Absent	1		MTT- Gland (s)	1.03 (0.94 ~ 1.13)	0.491
Present	0.4 (0.18 ~ 0.87)	**0.022**	MTT- Relative value (s)	0.96 (0.87 ~ 1.06)	0.421
CDFI flow grade			AUTIC- Nodule (AU·s)	0.74 (0.53 ~ 1.05)	**0.091**
None	1		AUTIC- Gland (AU·s)	0.89 (0.71 ~ 1.11)	0.304
Moderate	0.46 (0.21 ~ 1.01)	**0.054**	AUTIC- Relative value (AU·s)	0.94 (0.74 ~ 1.2)	0.643
Marked	1.14 (0.38 ~ 3.39)	0.817	AUWash in- Nodule (AU·s)	0.06 (0 ~ 2.25)	**0.127**
**SMI characteristics**			AUWash in- Gland (AU·s)	0.14 (0.01 ~ 1.66)	**0.12**
SMI flow grade			AUWash in- Relative value (AU·s)	2.06 (0.31 ~ 13.67)	0.454
None	1		AUWash out- Nodule (AU·s)	0.74 (0.51 ~ 1.06)	**0.1**
Moderate	0.66 (0.17 ~ 2.54)	0.543	AUWash out- Gland (AU·s)	0.88 (0.69 ~ 1.12)	0.31
Marked	0.69 (0.17 ~ 2.77)	0.596	AUWash out-Relative value (AU·s)	0.94 (0.71 ~ 1.23)	0.632
cSMI- VI (%)	1 (0.98 ~ 1.02)	0.979			

Abbreviations: OR odds ratio; CI confidence tnterval; CLNM cervical lymph node metastasis; PTC papillary thyroid carcinoma; BMI body mass index; US ultrasound; CDFI color doppler flow imaging; SMI superb microvascular imaging; cSMI-VI color superb microvascular imaging vascular index; mSMI-VI monochrome superb microvascular imaging vascular index; SWE shear wave elastography; SD standard devistion; CEUS contrast-enhanced ultrasound; AT arrival time; PI peak intensity; AU arbitrary units; TTP time to peak; MTT mean transit time; AUTIC area under the curve; AUWash-in wash-in area; AUWash-out wash-out area

### Screening of predictive variables of prediction models

**Fig. 3:**
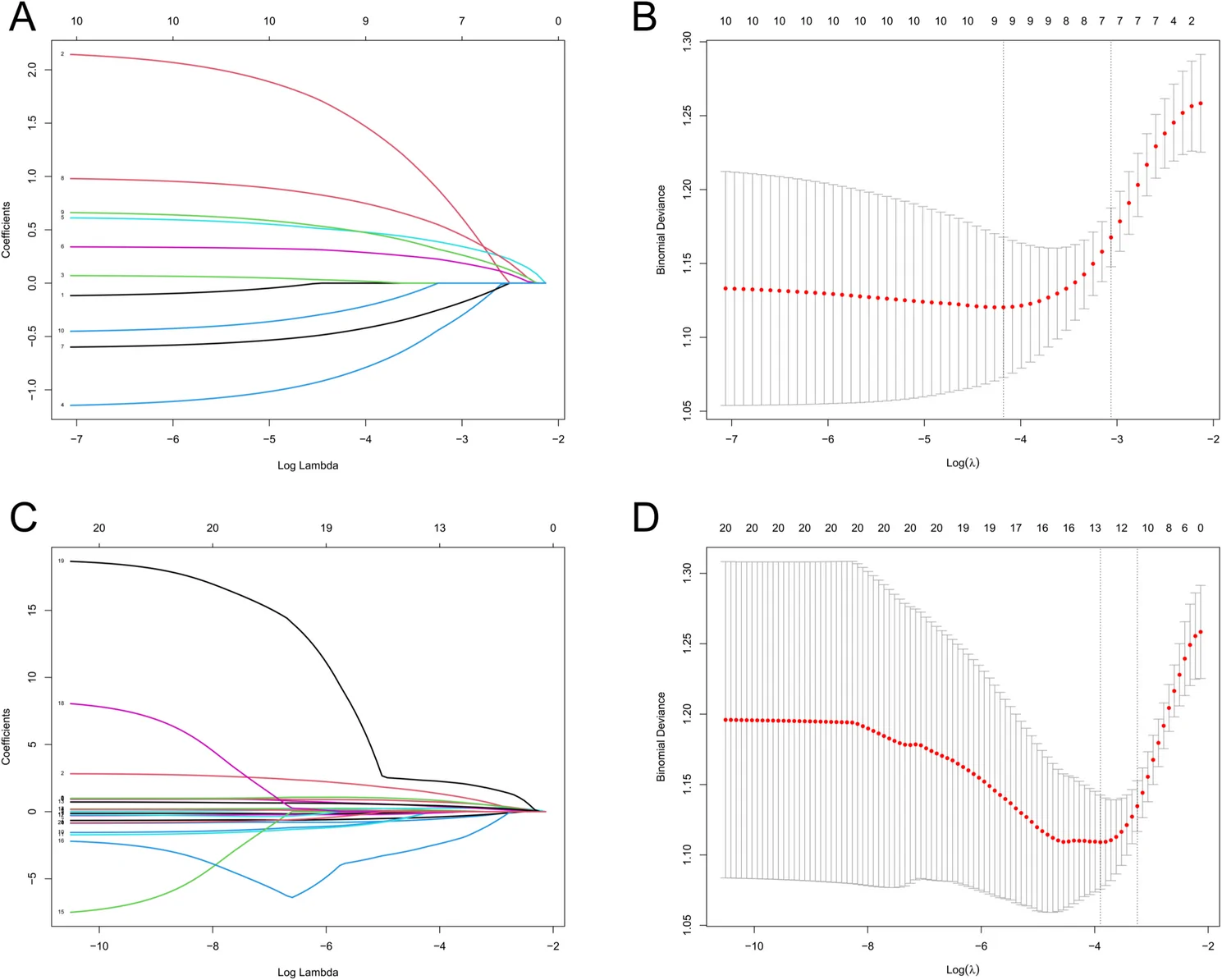
Feature selection using least absolute shrinkage and selection operator (LASSO) binary logistic regression for the conventional ultrasound model (**A**, **B**) and the multimodal ultrasound model (**C**, **D**)

To develop a robust predictive model, LASSO regression was applied to screen candidate variables with *P* < 0.20 in univariate analysis. Two models were constructed: a conventional ultrasound model integrating clinical and conventional US features, and a multimodal ultrasound model combining clinical and multimodal US features. The conventional model identified seven key predictors: shape, taller-than-wide sign, capsular relationship, capsular contact degree, diffuse background, multifocality, and size (Fig. 3A-B. The multimodal model yielded 12 predictors, including the seven conventional features plus microcalcification quantities, enhancement pattern, AT-nodule, AUWash-in-nodule, and SD-Gland (Fig. 3C-D). To assess SD-Gland stability, bootstrap-based LASSO analysis with 1,000 resamples was performed. SD-Gland was selected in 89.8% of bootstrap iterations with consistently positive coefficients, supporting the relative stability of this variable (Supplementary Table S1). These predictors were used to construct and visualize nomogram-based risk prediction models.

### Interobserver agreement

Based on 50 randomly selected thyroid nodule cases, two physicians independently interpreted the 12 ultrasound features finally included in the prediction models. The results showed the Cohen’s kappa and weighted Kappa values of all 8 categorical features ranged from 0.83 to 0.94, with the lower limit of 95% CI between 0.65 and 0.82, and the upper limit between 0.98 and 1.00; the ICC values of all 4 continuous features ranged from 0.93 to 0.99, with the lower limit of 95% CI between 0.87 and 0.98, and the upper limit between 0.96 and 0.99. All consistency coefficients were statistically significant (Table 3).

**Table 3:** Interobserver agreement for the 12 ultrasound features

Feature	Kappa/ICC coefficient(95% CI)	*P* value
Shape (Round or oval/ Irregular)	0.83 (0.65-1.00)	**< 0.01**
Taller than wide ( (Present/ absent )	0.91(0.78-1.00)	**< 0.01**
Multifocality (Absent/ present )	0.94 (0.82-1.00)	**< 0.01**
Diffuse background (Absent/ present )	0.88 (0.75-1.00)	**< 0.01**
Enhancement pattern (Centripetal/ Non-centripetal)	0.84(0.69–0.99)	**< 0.01**
US State with capsule (Distant/ Adjacent or invades the surroundings/ Unassessable)	0.89 (0.76-1.00)	**< 0.01**
Microcalcifications quantities (None/ <5/ ≥5)	0.90 (0.80–0.98)	**< 0.01**
US Contact capsule (< 25%/ 25% ~ 50%/ >50%)	0.84 (0.68–0.99)	**< 0.01**
Size (cm)	0.99 (0.98–0.99)	**< 0.01**
SD- Gland (m/s)	0.95(0.91–0.97)	**< 0.01**
AT- nodule (s)	0.93 (0.87–0.96)	**< 0.01**
AUWash in- Nodule(AU·s)	0.95(0.92–0.97)	**< 0.01**

Abbreviations: ICC intraclass correlation coefficient; CI confidence interval; US ultrasound; SD standard devistion; AT arrival time; AUWash-in wash-in area; AU arbitrary units

### Construction and validation of nomograms for predicting CLNM

Based on the weighted regression coefficients, two nomograms were constructed: a conventional ultrasound model and a multimodal ultrasound model. Internal validation was performed using the independent validation cohort (*n* = 61).

The predicted probability of CLNM was calculated using multivariable logistic regression equations. For the conventional ultrasound model, the prediction equation was as follows:

logit(p) = -4.166 + 2.446 × (Shape =” Irregular”)- 1.237 × (Taller than wide = “Absent”) + 0.969 × (US State with capsule = “Adjacent or invades the surroundings”) + 0.738 × (US State with capsule = “Unassessable”) + 0.253 × (US Contact capsule = “25% ~ 50%”) + 0.956 × (US Contact capsule = “>50%”)- 0.517 × (diffuse background = “Present”) + 1.020 × (Multifocality = “Present”) + 0.658 × Size( cm).

For the multimodal ultrasound model, the prediction equation was as follows:

logit(p) = -5.693 + 2.271 × (Shape = “Irregular”) + 0.523 × (Microcalcification quantities =”<5”) + 0.661 × (Microcalcification quantities =”≥5”)- 0.991 × (Taller than wide = “Absent”) + 0.794 × (US State with capsule = “Adjacent or invades the surroundings”)- 0.260 × (US State with capsule = “Unassessable”) + 0.093 × (US Contact capsule = “25% ~ 50%”) + 2.222 × (US Contact capsule =” >50%”)- 0.625 × (Diffuse background = “Present”) + 0.877 × (Multifocality = “Present”) + 1.254 × (Enhancement pattern = “Non-centripetal”) + 0.638 × Size(cm) + 0.062 × AT nodule(s)- 6.071 × AUWash in‑Nodule(AU·s) + 3.143 × SD‑Gland(m/s).

The predicted probability was calculated as:

$$P=\frac{1}{1+e^{-\operatorname{Logit}(P)}}$$

Detailed regression coefficients and variable coding are provided in Supplementary Tables S2 and S3.

**Fig. 4:**
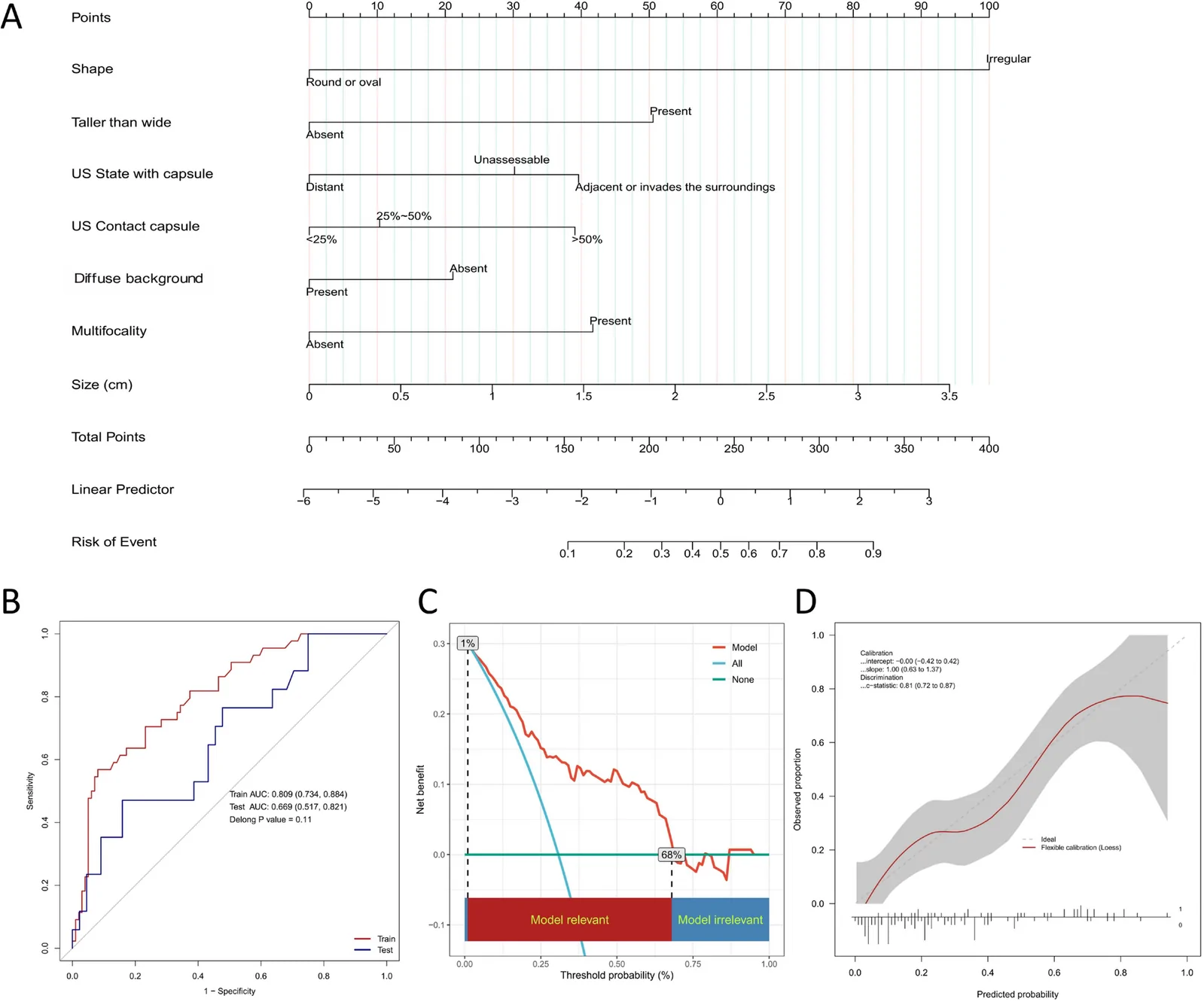
Development and validation of the conventional ultrasound nomogram for predicting occult cervical lymph node metastasis (CLNM): (**A**) Nomogram incorporating conventional ultrasound features. (**B**) Receiver operating characteristic (ROC) curves. (**C**) Decision curve analysis (DCA). (**D**) Calibration curve showing agreement between predicted and observed probabilities

The conventional ultrasound nomogram (Fig. 4A) achieved a bootstrap-corrected AUC of 0.809 (95% CI: 0.734–0.884) in the training cohort and 0.669 (95% CI: 0.517–0.821) in the validation cohort based on 500 bootstrap resamples (Fig. 4B). DeLong test showed no significant difference between the training and validation cohorts (*P* = 0.11). Decision curve analysis (DCA; Fig. 4C) demonstrated favorable net clinical benefit across threshold probabilities ranging from 0.01 to 0.68. Calibration curves (Fig. 4D) showed acceptable agreement between predicted and observed outcomes.

**Fig. 5:**
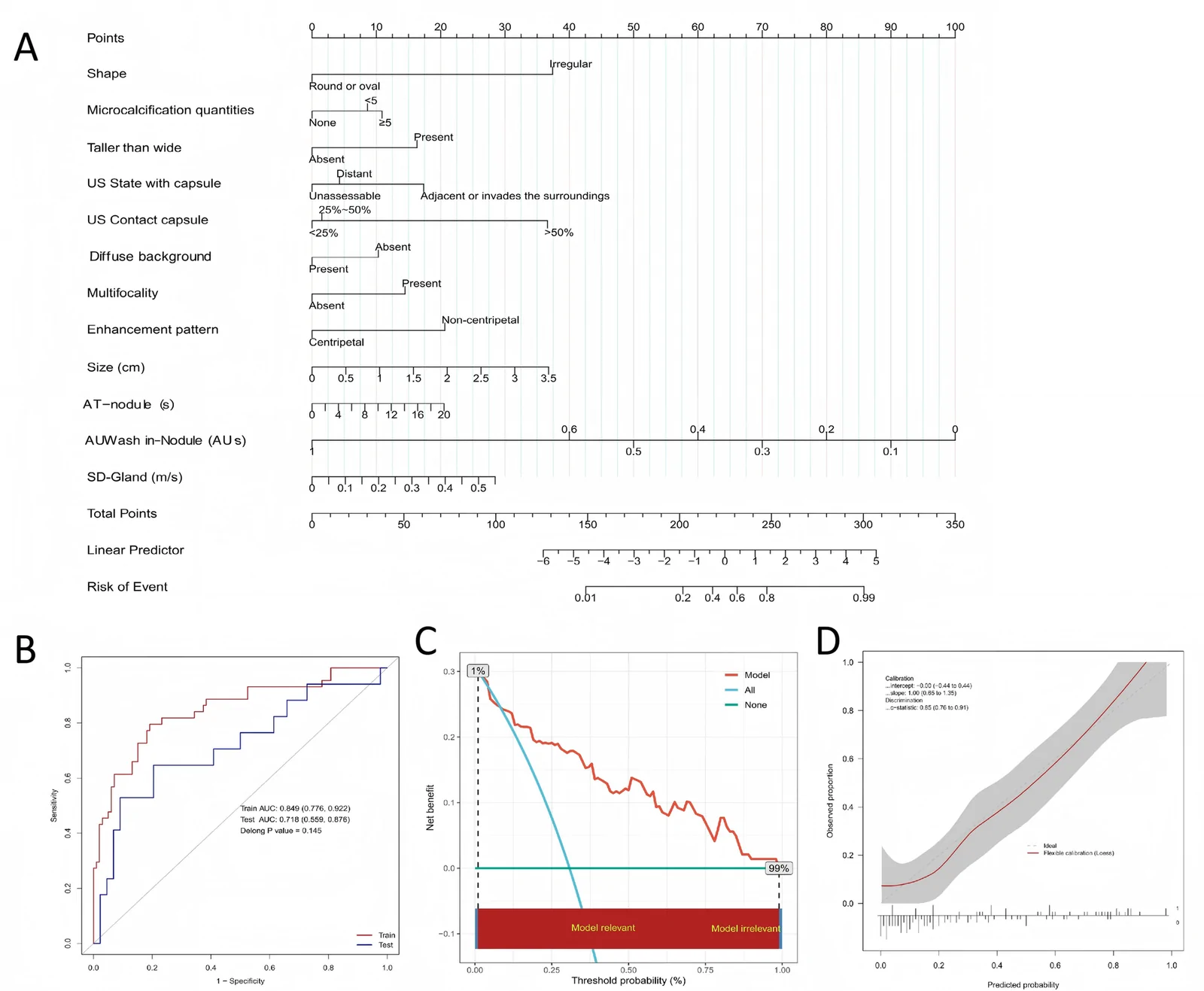
Development and validation of the multimodal ultrasound nomogram for predicting occult cervical lymph node metastasis (CLNM). (**A**) Nomogram incorporating conventional ultrasound, shear wave elastography (SWE) and contrast-enhanced ultrasound (CEUS) features. (**B**) Receiver operating characteristic (ROC) curves of the nomogram in the training and validation cohorts. (**C**) Decision curve analysis (DCA) of the nomogram. (**D**) Calibration curve showing agreement between predicted and observed probabilities

The multimodal ultrasound nomogram (Fig. 5A) achieved a bootstrap-corrected AUC of 0.849 (95% CI: 0.776–0.922) in the training cohort and 0.718 (95% CI: 0.559–0.876) in the validation cohort based on 500 bootstrap resamples (Fig. 5B). No significant difference was observed between the training and validation cohorts by DeLong test (*P* = 0.15). Compared with the conventional model, the multimodal model demonstrated improved discrimination and generalizability. DCA (Fig. 5C) showed consistent positive net clinical benefit across a wider threshold probability range (0.01–0.99). Calibration curves (Fig. 5D) demonstrated excellent concordance between predicted and observed probabilities of CLNM.

Direct comparison of the two models showed that the multimodal ultrasound model achieved higher AUCs than the conventional ultrasound model in both the training cohort (0.849 vs. 0.809) and validation cohort (0.718 vs. 0.669). However, these differences were not statistically significant in either the training cohort (DeLong test, *P* = 0.06) (Supplementary Figure **S1**) or validation cohort (DeLong test, *P* = 0.35) (Supplementary Figure **S2**). Overall, the multimodal model provided superior clinical utility and broader applicability according to DCA findings.

**Fig. 6:**
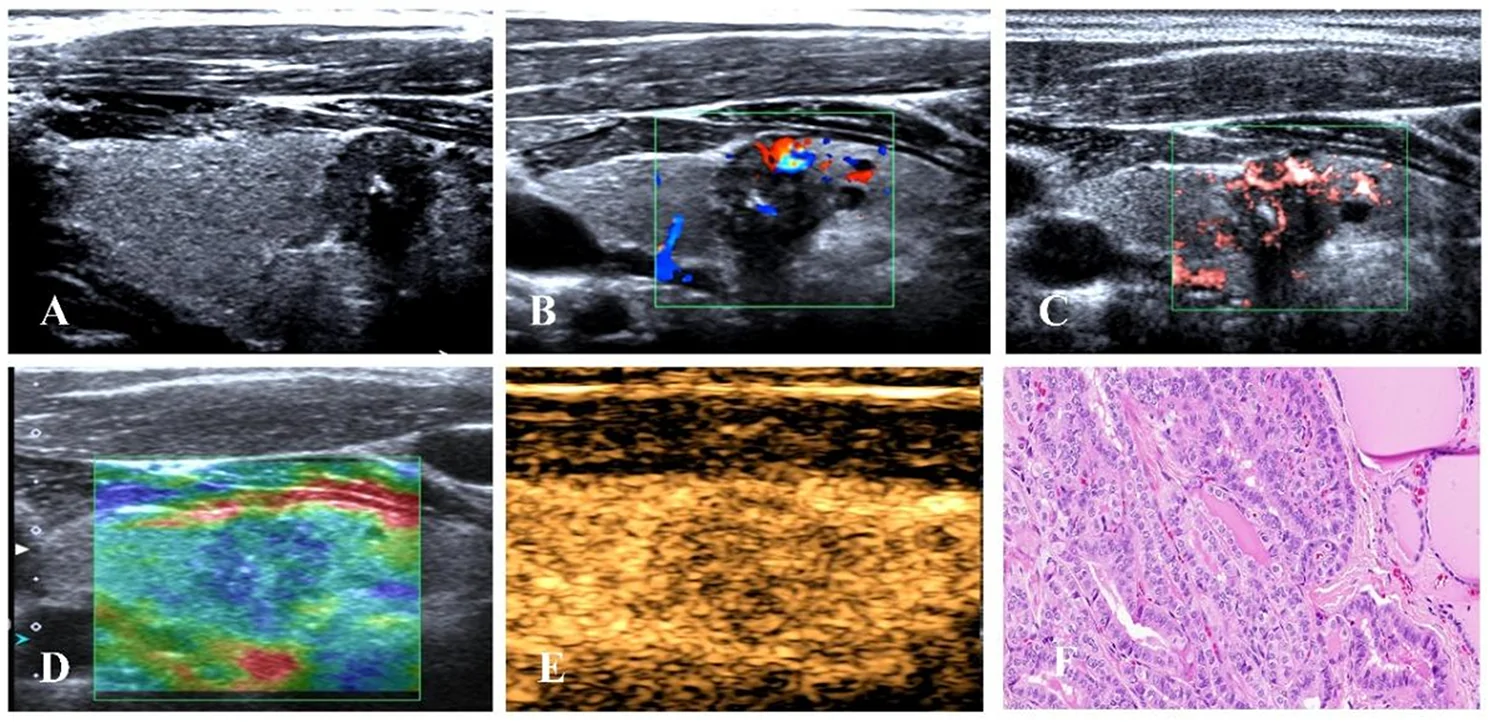
A male patient with postoperatively confirmed papillary thyroid carcinoma (PTC) in right thyroid lobe with central cervical lymph node metastasis (CLNM). (**A**) Grayscale ultrasound image of the nodule. (**B**) Color Doppler ultrasound image (CDFI) of the nodule. (**C**) Color superb microvascular imaging (cSMI) image. (**D**) Shear wave elastography (SWE) image, revealing that the nodule exhibits higher stiffness than the adjacent thyroid parenchyma. (**E**) Contrast-enhanced ultrasound (CEUS) image demonstrated that the nodule exhibits non-centripetal, heterogeneous hypo-enhancement. (**F**) Histopathological image of the nodule (HE×40)

A representative case is shown in Fig. 6. A 36-year-old man presented with bilateral thyroid nodules, with the highest TI-RADS category identified in a 1.5 cm nodule located in the right thyroid lobe. Conventional ultrasound demonstrated irregular morphology, microcalcifications (< 5), a taller-than-wide shape, and 25%–50% capsular contact without diffuse background changes (Fig. 6A). CDFI and SMI revealed increased intranodular vascularity (Fig. 6B, C). SWE showed that the lesion was stiffer than the surrounding thyroid parenchyma (Fig. 6D), with an SD-Gland value of 0.23 m/s. CEUS demonstrated heterogeneous hypo-enhancement with a non-centripetal enhancement pattern (Fig. 6E), with an AT-nodule of 19 s and an AUWash-in-Nodule of 0.2 AU·s. Based on these high-risk features, the multimodal nomogram estimated a high probability of CLNM. The patient subsequently underwent thyroidectomy with lymph node dissection, and postoperative histopathology confirmed PTC with CLNM (Fig. 6F). These findings further support the predictive performance and potential clinical utility of the multimodal nomogram.

**Fig. 7:**
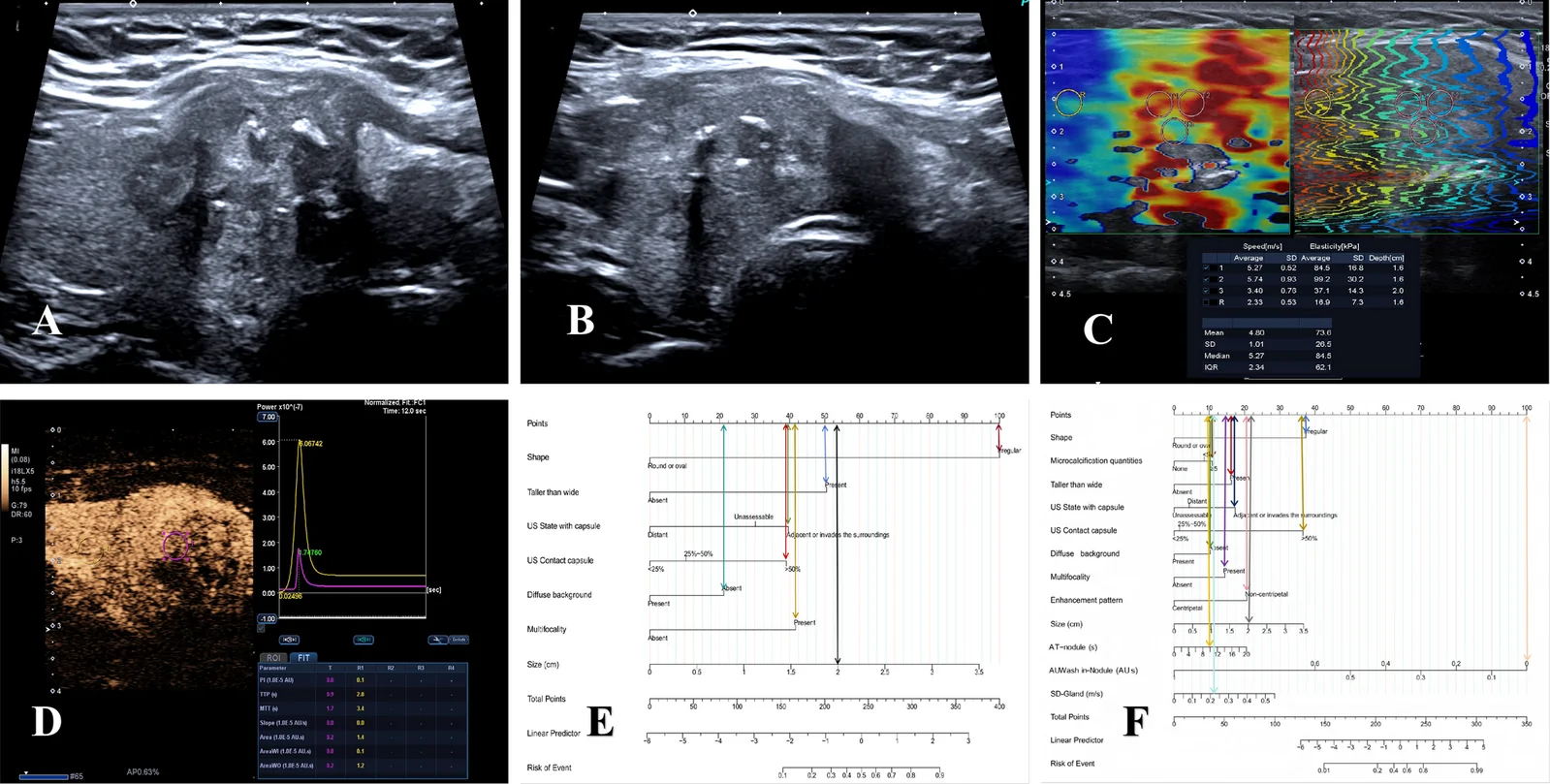
Practical application of the conventional and multimodal ultrasound nomograms in a patient with occult cervical lymph node metastasis (CLNM). (**A**, **B**) Grayscale ultrasound images demonstrate an irregular thyroid nodule with a taller-than-wide shape and more than 5 microcalcifications. The nodule is adjacent to the thyroid capsule, with capsular contact involving > 50% of both the anterior and posterior capsule. No diffuse thyroid parenchymal change is observed. The nodule measures approximately 2.0 cm, and an additional papillary thyroid carcinoma (PTC) lesion is identified in the contralateral lobe. (**C**) Shear wave elastography (SWE) image and quantitative analysis showing an SD-Gland value of 0.22 m/s. (**D**) Contrast-enhanced ultrasound (CEUS) image and quantitative analysis demonstrating a non-centripetal enhancement pattern, an AT-nodule value of 10 s, and an AUwash-in–nodule value of 0 AU·s. (**E**, **F**) Corresponding feature scores in the conventional ultrasound nomogram and multimodal ultrasound nomogram are indicated by arrows. The total scores are approximately 345 and 308, corresponding to predicted CLNM risks of > 90% and > 99%, respectively

**Fig. 8:**
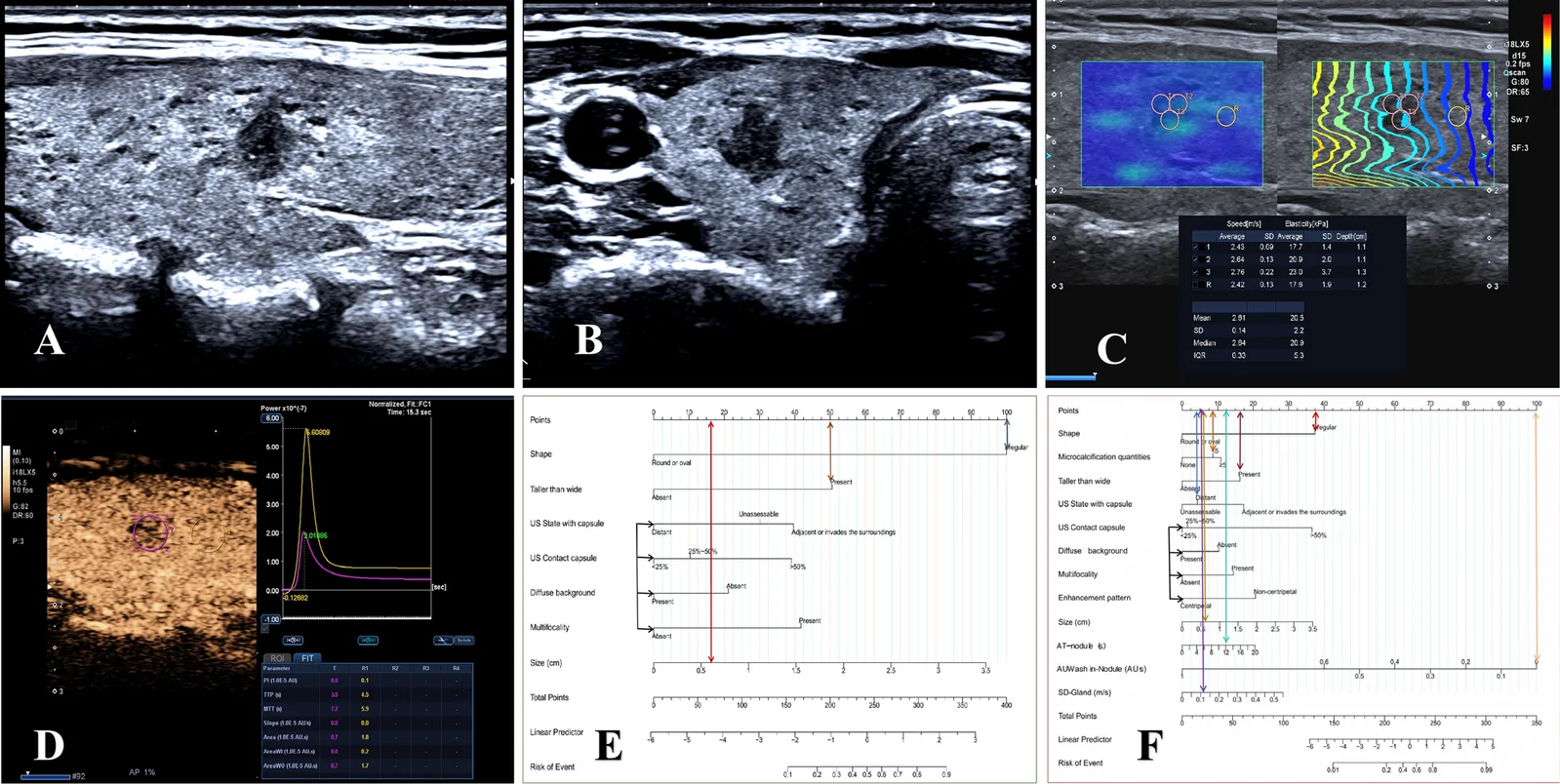
Practical application of the conventional and multimodal ultrasound nomograms in a patient without cervical lymph node metastasis (CLNM). (**A**, **B**) Grayscale ultrasound images demonstrate an irregular thyroid nodule with a taller-than-wide shape and fewer than 5 microcalcifications. The nodule is distant from the thyroid capsule, and diffuse thyroid parenchymal change is present in the background. The nodule measures approximately 0.6 cm, with no additional papillary thyroid carcinoma (PTC) lesions identified. (**C**) Shear wave elastography (SWE) image and quantitative analysis showing an SD-Gland value of 0.13 m/s. (**D**) Contrast-enhanced ultrasound (CEUS) image and quantitative analysis demonstrating a centripetal enhancement pattern, an AT-nodule value of 12 s, and an AU wash-in–nodule value of 0 AU·s. (E, F) Arrows indicate the corresponding feature scores in the conventional ultrasound nomogram and multimodal ultrasound nomogram. Black arrows indicate features assigned 0 points, whereas colored arrows indicate features assigned > 0 points. The total scores are approximately 166 and 191, corresponding to predicted CLNM risks of < 20% for both nomograms

To further demonstrate the clinical application and interpretability of the nomograms, one CLNM-positive case and one CLNM-negative case were evaluated using both the conventional ultrasound and multimodal ultrasound nomograms (Fig. 7 and Fig. 8). For each nomogram, points were assigned according to the category or value of each predictor. The total score was calculated by summing the individual points, and the corresponding probability of CLNM was obtained by projecting the total score onto the probability scale.

## Discussion

This study aimed to develop a multimodal ultrasound-based model for predicting occult cervical lymph node metastasis (CLNM) in clinically node-negative (cN0) papillary thyroid carcinoma (PTC). Accurate preoperative identification of occult CLNM is important for individualized treatment planning and surgical decision-making. The proposed multimodal ultrasound nomogram demonstrated good discrimination and calibration, achieving AUCs of 0.849 and 0.718 in the training and validation cohorts, respectively. Although the improvement in AUC over the conventional ultrasound model was not statistically significant in the validation cohort, decision curve analysis indicated greater net clinical benefit across a broad range of threshold probabilities, suggesting potential clinical value. Interobserver analysis demonstrated good reproducibility of the selected multimodal ultrasound features, which may be attributed to the radiologists’ extensive experience, standardized training, and predefined imaging protocol. These findings support the reproducibility of the selected ultrasound features. However, both radiologists were experienced operators from a high-volume medical center, and further validation among operators with different levels of expertise is needed to confirm the generalizability of the nomograms. These findings suggest that multimodal ultrasound may provide complementary information beyond conventional imaging for preoperative risk stratification. Several methodological characteristics of this study should also be noted. First, conventional ultrasonography was systematically combined with SMI, SWE, and CEUS to establish a multimodal assessment framework. Second, peritumoral stiffness heterogeneity (SD-Gland) was incorporated as a potential imaging biomarker reflecting the tumor microenvironment. Third, quantitative CEUS perfusion parameters were included to provide additional hemodynamic information beyond morphological assessment. Collectively, these approaches may improve individualized risk assessment in patients with cN0 PTC.

Previous studies mainly focused on single imaging modalities or qualitative feature combinations [19–22], with limited evaluation of peritumoral thyroid parenchymal characteristics. In contrast, our study integrated four ultrasound modalities—US, SMI, SWE and CEUS—to establish a comprehensive predictive model. Although SMI-derived parameters were not retained in the final model, this likely reflects cohort-specific characteristics rather than lack of biological relevance. The relatively small median tumor size in our cohort (0.7 cm) may have limited the detection of intratumoral microvascular signals by SMI, particularly in papillary thyroid microcarcinomas. Overall, the model incorporated morphological, hemodynamic, and biomechanical imaging features to better characterize tumor behavior.

Notably, peritumoral thyroid parenchymal features were included in the predictive framework. Diffuse thyroid disease was less common in the CLNM-positive group (*P* = 0.022), whereas perinodular glandular stiffness heterogeneity (SD-Gland) was significantly higher (*P* < 0.001); both variables were retained in the final LASSO model. Diffuse thyroid disease may exert a protective effect against PTC metastasis through modulation of the local immune microenvironment [23–25]. Conversely, increased SD-Gland may reflect microenvironmental remodeling associated with tumor invasion. Metastatic PTC cells can secrete cytokines and matrix-remodeling factors, including transforming growth factor-β1 and matrix metalloproteinases [26–28], which in turn may promote stromal fibrosis and desmoplastic reactions, thereby increasing local stiffness heterogeneity. Although SD-Gland was retained during LASSO-based feature selection, its wide confidence interval in univariate analysis suggested instability, likely due to limited sample size and skewed variable distribution. While SD-Gland provides novel insights into the role of the peritumoral microenvironment in PTC metastasis, it should be considered an exploratory biomarker at this stage that requires further validation and standardization before clinical application.

CEUS findings further supported the potential value of microenvironmental assessment. AT-nodule and AUWash-in-nodule were retained in the final model, and non-centripetal enhancement was significantly associated with CLNM. These findings suggest distinct microcirculatory characteristics in tumors with occult metastasis. Immature vascular architecture may contribute to prolonged rise time, whereas reduced effective vascular bed density may decrease overall perfusion volume [29–30]. This “fast-in, low-perfusion” pattern may reflect increased tumor invasiveness and highlights the additional value of CEUS in evaluating tumor angiogenesis. The exclusion of SMI-related parameters from the final model does not imply absence of predictive value. Possible explanations include: first, the small median tumor diameter (0.7 cm) in this cohort, with most lesions classified as microcarcinomas in which intratumoral neovascularization may not be detectable by SMI; second, CEUS-derived hemodynamic parameters may provide complementary biological information compared with microvascular morphology assessment. Instead, it may indicate that CEUS-derived hemodynamic parameters provided more informative or complementary biological information within this cohort. The mechanisms for excluding SMI parameters also require further exploration with larger samples and histopathological correlation.

Despite these encouraging findings, several limitations should be acknowledged. First, this was a single-center study conducted using a single ultrasound platform (Canon Aplio i900), and all image interpretations were performed by radiologists at our institution. The lower AUC in the validation cohort compared with the training cohort (0.718 vs. 0.849) suggests potential model instability. Although internal validation using random cohort splitting and bootstrap resampling indicated acceptable stability, independent external validation is still required. Future prospective multicenter studies with larger independent cohorts are warranted to externally validate our nomogram and further assess its clinical utility before broader clinical application. Second, SWE and CEUS are influenced by operator experience and equipment differences, which may limit generalizability, particularly in primary healthcare settings. Third, the relatively small sample size may increase the risk of overfitting. Although the sample size was moderate, strict internal validation via bootstrap resampling and LASSO regression confirmed the model’s stability and reduced overfitting risk, the risk of overfitting cannot be completely eliminated. More clinical trials and external validation in multicenter cohorts is warranted to confirm generalizability. Only surgically treated cN0 PTC patients were included; therefore, potential selection and verification biases cannot be excluded. This study excluded those with preoperative evidence of CLNM, who differed significantly from the included patients in several baseline characteristics (Supplementary Table S4). In addition, non-surgical cN0 PTC patients were excluded, thereby excluding small or low-risk PTC nodules managed conservatively. Patients with incomplete preoperative multimodal ultrasound were also excluded; while baseline comparisons showed no significant differences (Supplementary Table S5), small low-risk lesions may lack complete multimodal imaging. These factors may introduce selection bias and potential verification bias.This study should therefore be regarded as an exploratory model development study.

The proposed multimodal ultrasound nomogram may serve as a visual decision-support tool for individualized management of cN0 PTC. Patients identified as high risk for occult CLNM may benefit from more aggressive surgical evaluation, whereas low-risk patients may avoid unnecessary surgical trauma and related complications. However, the current findings should be interpreted cautiously because residual selection bias and cohort-specific imaging characteristics may still influence model performance. Future studies should focus on external validation of the proposed nomogram using independent multicenter cohorts. In particular, the incremental predictive value of the newly identified multimodal ultrasound features, such as CEUS-related parameters, and peritumoral thyroid stiffness heterogeneity, warrants further investigation in larger prospective studies. Furthermore, artificial intelligence-assisted analysis may help improve the standardization and reproducibility of multimodal ultrasound assessment.

## Conclusion

In this study, we developed and internally validated a multimodal ultrasound nomogram integrating conventional ultrasound, SWE, and CEUS features to predict occult CLNM in patients with cN0 PTC. The multimodal framework incorporated both intratumoral imaging characteristics and peritumoral stiffness heterogeneity (SD-Gland), together with quantitative CEUS perfusion parameters, to provide complementary information beyond conventional imaging assessment. In this single-center cohort, the multimodal model achieved numerically higher AUCs than the conventional ultrasound model in both the training and validation cohorts, although the differences were not statistically significant. DCA suggested a greater potential clinical benefit across a wider range of threshold probabilities.This visualized risk stratification tool may facilitate individualized decision-making regarding prophylactic central neck dissection. Although model performance declined slightly in the validation cohort, the nomogram maintained acceptable discrimination and calibration. Further large-scale multicenter prospective studies with standardized imaging protocols are needed to validate its robustness and generalizability.

## Supplementary Information

Below is the link to the electronic supplementary material.

Supplementary Material 1

Supplementary Material 2

Supplementary Material 3

## Data Availability

The datasets generated or analyzed during the study are available from the corresponding author upon reasonable request.

## Abbreviations

ATA: American Thyroid Association
AT: Arrival Time
AUC: Area Under the Curve
AU: Arbitrary Units
AUWash-in: Wash-in Area Under the Curve
AUWash-out: Wash-out Area Under the Curve
AUTIC: Area Under the Time-Intensity Curve
BMI: Body Mass Index
BRAF: v-raf murine sarcoma viral oncogene homolog B1
CDFI: Color Doppler Flow Imaging
CEUS: Contrast-Enhanced Ultrasound
CI: Confidence Interval
CLNM: Cervical Lymph Node Metastasis
cSMI: color Superb Microvascular Imaging
CT: Computed Tomography
DCA: Decision Curve Analysis
ICC: Intraclass Correlation Coefficient
LASSO: Least Absolute Shrinkage and Selection Operator
mSMI: monochrome Superb Microvascular Imaging
MI: Mechanical Index
MTT: Mean Transit Time
OR: Odds Ratio
pCND: prophylactic Central Neck Dissection
PI: Peak Intensity
PTC: Papillary Thyroid Carcinoma
PTMC: Papillary Thyroid Microcarcinoma
ROC: Receiver Operating Characteristic
ROI: Region of Interest
SD: Standard Deviation
SMI: Superb Microvascular Imaging
SWE: Shear Wave Elastography
TGF-β1: Transforming Growth Factor Beta 1
TIC: Time-Intensity Curve
TI-RADS: Thyroid Imaging Reporting and Data System
TTP: Time to Peak
US: Ultrasonography
VI: Vascular Index
