# Supplementary material for: Multimodal ultrasound-based nomogram for predicting occult cervical lymph node metastasis in cN0 papillary thyroid carcinoma
Source: BMC Med Imaging. 2026 Aug 10;26:587. doi: 10.1186/s12880-026-02648-x (PMC13737597; doi:10.1186/s12880-026-02648-x)
Supplement: Supplementary file 2 [file 12880_2026_2648_MOESM3_ESM.pdf]

TRIPOD+AI for Abstracts

Section and item	Checklist item	Reported on Page
**Title**		
1	Identify the study as developing or evaluating the performance of a multivariable prediction model, the target population, and the outcome to be predicted	1
**Background**		
2	Provide a brief explanation of the healthcare context and rationale for developing or evaluating the performance of all models	1-2
**Objectives**		
3	Specify the study objectives, including whether the study describes model development, evaluation, or both	1
**Methods**		
4	Describe the sources of data	1
5	Describe the eligibility criteria and setting where the data were collected	Not repoeted
6	Specify the outcome to be predicted by the model, including time horizon of predictions in case of prognostic models	2
7	Specify the type of model, a summary of the model-building steps, and the method for internal validation†	1-2
8	Specify the measures used to assess model performance (eg, discrimination, calibration, clinical utility)	2
**Results**		
9	Report the number of participants and outcome events	1-2
10	Summarise the predictors in the final model†	2
11	Report model performance estimates (with confidence intervals)	2
**Discussion**		
12	Give an overall interpretation of the main results	2
**Registration**		
13	Give the registration number and name of the registry or repository	Not Reported
