# Supplementary material for: Multimodal ultrasound-based nomogram for predicting occult cervical lymph node metastasis in cN0 papillary thyroid carcinoma
Source: BMC Med Imaging. 2026 Aug 10;26:587. doi: 10.1186/s12880-026-02648-x (PMC13737597; doi:10.1186/s12880-026-02648-x)
Supplement: Supplementary file 3 [file 12880_2026_2648_MOESM1_ESM.docx]

# Supplementary Material

**Manuscript Title:** Multimodal Ultrasound-Based Nomogram for Predicting Occult Cervical Lymph Node Metastasis in cN0 Papillary Thyroid Carcinoma

**Yukang Zhang ^1^, Yang Guang ^2^, Sen Wang ^1^, Wen He ^1^*, Wei Zhang ^1^***

^1^ Department of Ultrasound, Beijing Tiantan Hospital, Capital Medical University, Beijing 100070, China

^2^ Department of Ultrasound, Beijing Electric Power Hospital of State Grid Corporation of China, Beijing 100073, China

**Manuscript ID:** a8401b23 -d476-49b9-a769-6ccf88a13c82

# **Table of Contents**

1. Supplementary Tables

·Supplementary Table S1. Bootstrap-based LASSO stability selection and variable rank results ...........................................................................................................Page 2

·Supplementary Table S2. Regression coefficients and variable coding of the conventional ultrasound model for predicting CLNM......................................... Page 3

·Supplementary Table S3. Regression coefficients and variable coding of the multimodel ultrasound model for predicting CLNM.................................................................... Page 4

·Supplementary Table S4. Baseline clinical and ultrasound characteristics: included vs. excluded patients with preoperative imaging suspicious for CLNM................................................................................................................Page 5-6

·Supplementary Table S5. Baseline clinical and ultrasound characteristics: included vs. excluded patients with incomplete multimodal ultrasound data ...................................................................................................................... Page 7

2. Supplementary Figures

·Supplementary Figure S1. Receiver operating characteristic (ROC) curve comparison between the conventional ultrasound model and multimodal ultrasound model in the training cohort using the DeLong test. ........................................... Page 8

·Supplementary Figure S2. Receiver operating characteristic (ROC) curve comparison between the conventional ultrasound model and multimodal ultrasound model in the validation cohort using the DeLong test.......................................... Page 9

**Supplementary Table S1. Bootstrap-based LASSO stability selection and**

**variable rank results**

| **LASSO Stability Selection Results — Bootstrap B=1000** | | | | | | | |
| --- | --- | --- | --- | --- | --- | --- | --- |
| **Bootstrap resamples** | 1000.000 | **Random seed** | 20260517.000 | **Primary candidate variables** | 64.000 | **Uploaded Lasso.var.min variables** | 12.000 |
| **Primary CV-selected C** | 0.160 | **Primary lambda=1/C** | 6.251 | **Primary apparent AUC** | 0.816 | **Mean selected/boot** | 18.810 |
| **Conditional C** | 0.494 | **Conditional lambda=1/C** | 2.024 | **Conditional apparent AUC** | 0.813 | **Mean selected/boot** | 10.884 |
|  |  |  |  |  |  |  |  |
| **Variable** | **Analysis** | **Selected_count_B1000** | **Selection_probability_B1000** | **Positive_probability** | **Negative_probability** | **Sign_stability_among_selected** |  |
| SD-Gland (m/s) | Primary full-candidate | 898.000 | 0.898 | 0.898 | 0.000 | 1.000 |  |
| SD-Gland (m/s) | Conditional uploaded 12 vars | 939.000 | 0.939 | 0.928 | 0.011 | 0.988 |  |
| **Rank** | **Variable** | **Selected_count_B1000** | **Selection_probability_B1000** | **In_uploaded_Lasso.var.min** | **Selected_in_original_CV_LASSO** | **Mean_bootstrap_coef** | **Sign_stability_among_selected** |
| 1 | Enhancement pattern | 912 | 0.912 | TRUE | TRUE | 0.264 | 1.000 |
| 2 | SD-Gland (m/s) | 898 | 0.898 | TRUE | TRUE | 0.235 | 1.000 |
| 3 | US State with capsule | 873 | 0.873 | TRUE | TRUE | 0.219 | 1.000 |
| 4 | Size | 873 | 0.873 | TRUE | TRUE | 0.215 | 1.000 |
| 5 | Shape | 869 | 0.869 | TRUE | TRUE | 0.180 | 1.000 |
| 6 | Diffuse background | 863 | 0.863 | TRUE | TRUE | -0.186 | 1.000 |
| 7 | Multifocality | 854 | 0.854 | TRUE | TRUE | 0.179 | 1.000 |
| 8 | Taller than wide | 753 | 0.753 | TRUE | TRUE | -0.155 | 0.999 |
| 9 | Microcalcification quantities | 690 | 0.690 | TRUE | TRUE | 0.124 | 0.999 |
| 10 | Age | 687 | 0.687 | FALSE | TRUE | -0.118 | 1.000 |

Notes: Only the top 10 variables with a selection probability >0.60 are presented.

Abbreviations: CLNM, central lymph node metastasis; CV, cross-validation; LASSO, least absolute shrinkage and selection operator; AUC, area under the receiver operating characteristic curve; Coef, coefficient.

## Supplementary Table S2. Regression coefficients and variable coding of the conventional ultrasound model for predicting CLNM

| **Predictor** | **Coding** | **β coefficient** | **SE** | **Z value** | **P value** |
| --- | --- | --- | --- | --- | --- |
| (Intercept) | — | -4.166 | 1.117 | -3.729 | <0.001 |
| Shape | Round or oval (reference) | — | — | — | — |
|  | Irregular | 2.446 | 0.966 | 2.532 | 0.011 |
| Taller than wide | Present (reference) |  |  |  |  |
|  | Absent | -1.237 | 0.458 | -2.7 | 0.007 |
| US State with capsule | Distant (reference) | — | — | — | — |
|  | Adjacent or invades the surroundings | 0.969 | 0.532 | 1.821 | 0.069 |
|  | Unassessable | 0.738 | 1.128 | 0.655 | 0.513 |
| US Contact capsule | <25% (reference) | — | — | — | — |
|  | 25% ~ 50% | 0.253 | 0.575 | 0.44 | 0.66 |
|  | >50% | 0.956 | 0.912 | 1.048 | 0.295 |
| diffuse background | Absent (reference) | — | — | — | — |
|  | Present | -0.517 | 0.465 | -1.112 | 0.266 |
| Multifocality | Absent (reference) | — | — | — | — |
|  | Present | 1.02 | 0.447 | 2.28 | 0.023 |
| Size (cm) |  | 0.658 | 0.314 | 2.093 | 0.036 |

*Abbreviations: CLNM* cervical lymph node metastasis; *US* ultrasound

## Supplementary Table S3. Regression coefficients and variable coding of the multimodel ultrasound model for predicting CLNM

| **Predictor** | **Coding** | **β coefficient** | **SE** | **Z value** | **P value** |
| --- | --- | --- | --- | --- | --- |
| (Intercept) | — | -5.693 | 1.696 | -3.357 | 0.001 |
| Shape | Round or oval (reference) | — | — | — | — |
|  | Irregular | 2.271 | 1.104 | 2.057 | 0.04 |
| Microcalcification quantities | None (reference) | — | — | — | — |
|  | <5 | 0.523 | 0.656 | 0.798 | 0.425 |
|  | ≥5 | 0.661 | 0.565 | 1.171 | 0.242 |
| Taller than wide | Present (reference) | — | — | — | — |
|  | Absent | -0.991 | 0.498 | -1.988 | 0.047 |
| US State with capsule | Distant (reference) | — | — | — | — |
|  | Adjacent or invades the surroundings | 0.794 | 0.577 | 1.376 | 0.169 |
|  | Unassessable | -0.26 | 1.367 | -0.19 | 0.849 |
| US Contact capsule | <25% (reference) | — | — | — | — |
|  | 25% ~ 50% | 0.093 | 0.643 | 0.144 | 0.885 |
|  | >50% | 2.222 | 1.264 | 1.759 | 0.079 |
| diffuse background | Absent (reference) | — | — | — | — |
|  | Present | -0.625 | 0.526 | -1.189 | 0.234 |
| Multifocality | Absent (reference) | — | — | — | — |
|  | Present | 0.877 | 0.496 | 1.769 | 0.077 |
| Enhancement pattern | Centripetal (reference) | — | — | — | — |
|  | Non-centripetal | 1.254 | 0.524 | 2.394 | 0.017 |
| Size(cm) |  | 0.638 | 0.33 | 1.935 | 0.053 |
| AT-nodule(s) |  | 0.062 | 0.098 | 0.638 | 0.524 |
| AUWash in-Nodule(AU·s) |  | -6.071 | 3.017 | -2.012 | 0.044 |
| SD-Gland(m/s) |  | 3.143 | 3.035 | 1.036 | 0.3 |

*Abbreviations: CLNM* cervical lymph node metastasis; *US* ultrasound; *AT* arrival time; *AUWash-in* wash-in area; *SD* standard devistion

**Supplementary Table S4. Baseline clinical and ultrasound characteristics: included vs. excluded patients with preoperative imaging suspicious for CLNM**

| **Variables** | **Total**  **(n = 327)** | **The included patients**  **(n = 204)** | **The excluded patients with preoperative imaging suspicious for CLNM**  **(n = 123)** | ***P* value** |
| --- | --- | --- | --- | --- |
| Gender, n (%) |  |  |  | 0.299^a^ |
| Female | 247 (75.5) | 158 (77.5) | 89 (72.4) |  |
| Male | 80 (24.5) | 46 (22.5) | 34 (27.6) |  |
| Shape, n (%) |  |  |  | 0.053^a^ |
| Round or oval | 32 ( 9.8) | 25 (12.3) | 7 (5.7) |  |
| Irregular | 295 (90.2) | 179 (87.7) | 116 (94.3) |  |
| Microcalcifications quantities, n (%) |  |  |  | **0.001^a^** |
| None | 115 (35.2) | 82 (40.2) | 33 (26.8) |  |
| <5 | 76 (23.2) | 53 (26) | 23 (18.7) |  |
| ≥5 | 136 (41.6) | 69 (33.8) | 67 (54.5) |  |
| Echogenicity, n (%) |  |  |  | 0.773^b^ |
| Hyperechoic or isoechoic | 13 ( 4.0) | 9 (4.4) | 4 (3.3) |  |
| Hypoechoic | 314 (96.0) | 195 (95.6) | 119 (96.7) |  |
| Taller than wide, n (%) |  |  |  | **0.008^a^** |
| Present | 190 (58.1) | 107 (52.5) | 83 (67.5) |  |
| Absent | 137 (41.9) | 97 (47.5) | 40 (32.5) |  |
| US State with capsule, n (%) |  |  |  | **< 0.001^b^** |
| Distant | 114 (34.9) | 87 (42.6) | 27 (22) |  |
| Adjacent or invades the surroundings | 203 (62.1) | 111 (54.4) | 92 (74.8) |  |
| Unassessable | 10 ( 3.1) | 6 (2.9) | 4 (3.3) |  |
| Diffuse background, n (%) |  |  |  | 0.157^a^ |
| Absent | 202 (61.8) | 120 (58.8) | 82 (66.7) |  |
| Present | 125 (38.2) | 84 (41.2) | 41 (33.3) |  |
| Multifocality, n (%) |  |  |  | 0.73^a^ |
| Absent | 198 (60.6) | 125 (61.3) | 73 (59.3) |  |
| Present | 129 (39.4) | 79 (38.7) | 50 (40.7) |  |
| Age, Mean ± SD | 42.6 ± 12.2 | 43.7 ± 11.8 | 40.7 ± 12.7 | **0.031^c^** |
| Size, Median (IQR) | 0.8 (0.6, 1.2) | 0.7 (0.6, 1.0) | 1.1 (0.8, 1.6) | **< 0.001^d^** |
| BMI, Mean ± SD | 24.6 ± 3.9 | 24.8 ± 4.3 | 24.3 ± 3.2 | 0.34^c^ |

*Abbreviations: CLNM* cervical lymph node metastasis; *US* ultrasound; *SD* standard devistion; *IQR* interquartile range;*BMI* body mass index

Note: P-values with superscript a by Pearson’s Chi-square test; P-values with superscript b by Fisher’s exact test; P-values with superscript c were calculated by independent-samples t test; P-values with superscript d by Mann-Whitney U test.

**Supplementary Table S5. Baseline clinical and ultrasound characteristics: included vs. excluded patients with incomplete multimodal ultrasound data**

| **Variables** | **Total**  **(n = 318)** | **The included patients**  **(n = 204)** | **The excluded patients with incomplete multimodal ultrasound data**  **(n = 114)** | ***P*** |
| --- | --- | --- | --- | --- |
| Gender, n (%) |  |  |  | 0.817^a^ |
| Female | 245 (77.0) | 158 (77.5) | 87 (76.3) |  |
| Male | 73 (23.0) | 46 (22.5) | 27 (23.7) |  |
| Shape, n (%) |  |  |  | 0.082^a^ |
| Round or oval | 32 (10.1) | 25 (12.3) | 7 (6.1) |  |
| Irregular | 286 (89.9) | 179 (87.7) | 107 (93.9) |  |
| Echogenicity, n (%) |  |  |  | 0.548^b^ |
| Hyperechoic or isoechoic | 12 ( 3.8) | 9 (4.4) | 3 (2.6) |  |
| Hypoechoic | 306 (96.2) | 195 (95.6) | 111 (97.4) |  |
| Taller than wide, n (%) |  |  |  | 0.105^a^ |
| Present | 156 (49.1) | 107 (52.5) | 49 (43) |  |
| Absent | 162 (50.9) | 97 (47.5) | 65 (57) |  |
| US State with capsule, n (%) |  |  |  | 0.162^b^ |
| Distant | 134 (42.1) | 87 (42.6) | 47 (41.2) |  |
| Adjacent or invades the surroundings | 178 (56.0) | 111 (54.4) | 67 (58.8) |  |
| Unassessable | 6 ( 1.9) | 6 (2.9) | 0 (0) |  |
| Diffuse background, n (%) |  |  |  | 0.653^a^ |
| Absent | 190 (59.7) | 120 (58.8) | 70 (61.4) |  |
| Present | 128 (40.3) | 84 (41.2) | 44 (38.6) |  |
| Multifocality, n (%) |  |  |  | 0.86^a^ |
| Absent | 196 (61.6) | 125 (61.3) | 71 (62.3) |  |
| Present | 122 (38.4) | 79 (38.7) | 43 (37.7) |  |
| Age, Mean ± SD | 44.0 ± 11.8 | 43.7 ± 11.8 | 44.6 ± 11.9 | 0.529^c^ |
| BMI, Mean ± SD | 24.8 ± 4.1 | 24.8 ± 4.3 | 24.9 ± 3.8 | 0.762^c^ |
| Size, Mean ± SD | 0.9 ± 0.6 | 0.9 ± 0.7 | 0.8 ± 0.4 | 0.065^c^ |

*Abbreviations: US* ultrasound; *SD* standard devistion; *BMI* body mass index

Note: P-values with superscript a by Pearson’s Chi-square test; P-values with superscript b by Fisher’s exact test; P-values with superscript c were calculated by independent-samples t test.

Supplementary Figure S1. Receiver operating characteristic (ROC) curve comparison between the conventional ultrasound model and multimodal ultrasound model in the training cohort using the DeLong test.

Supplementary Figure S2. Receiver operating characteristic (ROC) curve comparison between the conventional ultrasound model and multimodal ultrasound model in the validation cohort using the DeLong test.
